# Supplementary material for: 3D RNA-scaffolded wireframe origami
Source: Nat Commun. 2023 Jan 24;14:382. doi: 10.1038/s41467-023-36156-1 (PMC9872083; doi:10.1038/s41467-023-36156-1)
Supplement: Supplementary file 1 — Supplementary Information [file 41467_2023_36156_MOESM1_ESM.pdf]

Supplementary Information

3D RNA-scaffolded wireframe origami

Molly F. Parsons[1], Matthew F. Allan[1-3], Shanshan Li[4,†], Tyson R. Shepherd[1], Sakul Ratanalert[1,5,‡], Kaiming Zhang[4,†], Krista M. Pullen[1], Wah Chiu[4,6], Silvi Rouskin[2], Mark Bathe[1]*

[1]Department of Biological Engineering, Massachusetts Institute of Technology, Cambridge, MA 02139, United States
[2]Department of Microbiology, Harvard Medical School, Boston, MA, United States
[3]Computational and Systems Biology, Massachusetts Institute of Technology, Cambridge, MA 02139, United States
[4]Department of Bioengineering, Stanford University, Stanford, CA 94305, United States
[5]Department of Chemical Engineering, Massachusetts Institute of Technology, Cambridge, MA 02139, United States
[6]CryoEM and Bioimaging Division, Stanford Synchrotron Radiation Lightsource, SLAC National Accelerator Laboratory, Stanford University, Menlo Park, CA 94025, United States

[†]*Present address*: MOE Key Laboratory for Cellular Dynamics and Division of Life Sciences and Medicine, University of Science and Technology of China, Hefei 230027, China
[‡]*Present address:* Inscripta, Inc., Boulder, CO 80027
[§]*Present address:* Department of Chemical and Biomolecular Engineering, Johns Hopkins University, Baltimore, MD 21218, United States

These authors contributed equally: Molly F. Parsons, Matthew F. Allan, Shanshan Li

*Correspondence to: mark.bathe@mit.edu

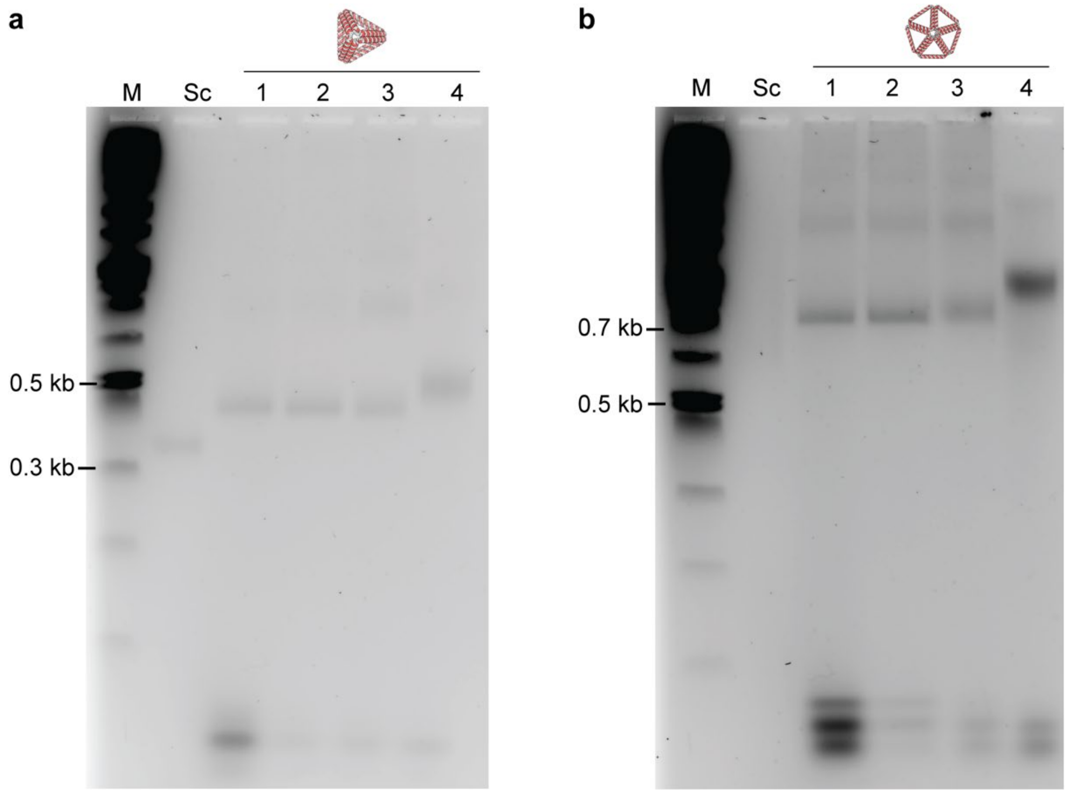

Supplementary Figure 1. **Comparison of protocols for folding RNA-scaffolded 3D DX wireframe origami.** Protocols compared were 20 nM scaffold in **1.** 10 mM HEPES pH 7.5 with 300 mM KCl, annealed using an overnight folding ramp (adapted from that used for analogous B-form DX wireframe origami[1]) with 20x molar excess of staples; **2.** Same as previous but with 10x staple excess; **3.** 40 mM Tris pH 8.0 with 20 mM acetic acid, 2 mM EDTA, and 12.5 mM magnesium acetate, annealed using a 40 min folding ramp with 10x molar excess of staples (protocol from Wang et al[2]); and **4.** 5 mM Tris pH 7.5 with 1 mM EDTA and 40 mM NaCl, annealed using an overnight folding ramp with 10x molar excess of staples (protocol from Zhou et al[3]). **a** Gel mobility shift assay of an EGFP mRNA-scaffolded tetrahedron with six helical turns (66 bp) per edge, *n* = 1 replicate. **b** Gel mobility shift assay of a 23s rRNA-scaffolded pentagonal bipyramid with six helical turns (66 bp) per edge, *n* = 1 replicate. The “Sc” lanes show the respective scaffold RNA not folded with staples. The high degree of internal structure in the 23s rRNA scaffold often results in a diffuse band in these non-denaturing agarose gels containing 2 mM magnesium acetate. “M” refers to the 1 kb Plus DNA Ladder (NEB) used as a marker for molecular weight.

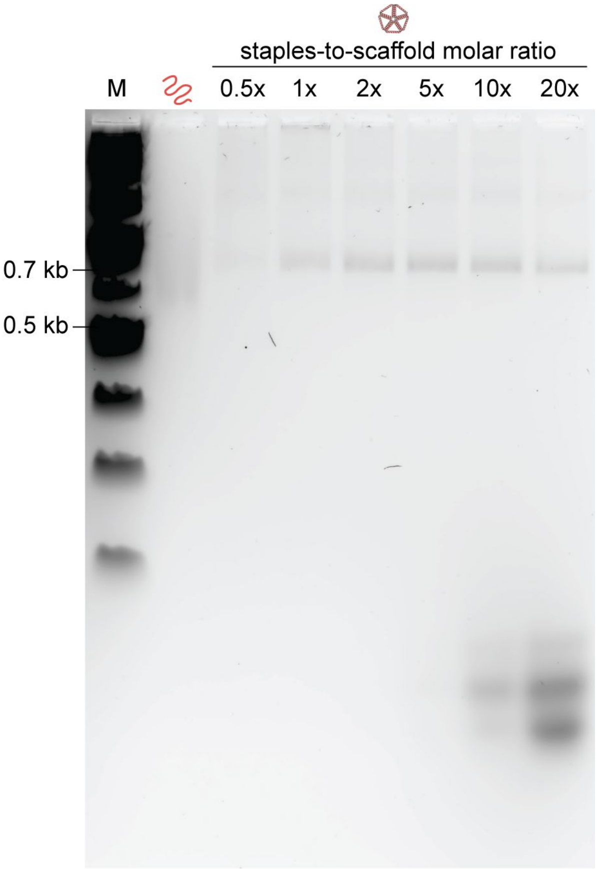

Supplementary Figure 2. **Gel mobility shift assay for A-form rPB66 folded with varying molar ratios of staples to scaffold.** The shift of the folded rPB66 band appears to stabilize at approximately 2x staples-to-scaffold ratio, suggesting this is the minimum amount of staples to use for proper folding, $n = 1$ replicate. A 1x staples-to-scaffold molar ratio led to a greater upward shift of the folded band relative to the scaffold band, as well as a broader band and more apparent aggregation, suggesting only partial folding, or perhaps reduced yield of correct structures. The reduced staples-to-scaffold ratio requirement for RNA:DNA origami relative to DNA:DNA origami is likely due to the higher affinity in the hybrid duplexes of the former[2].

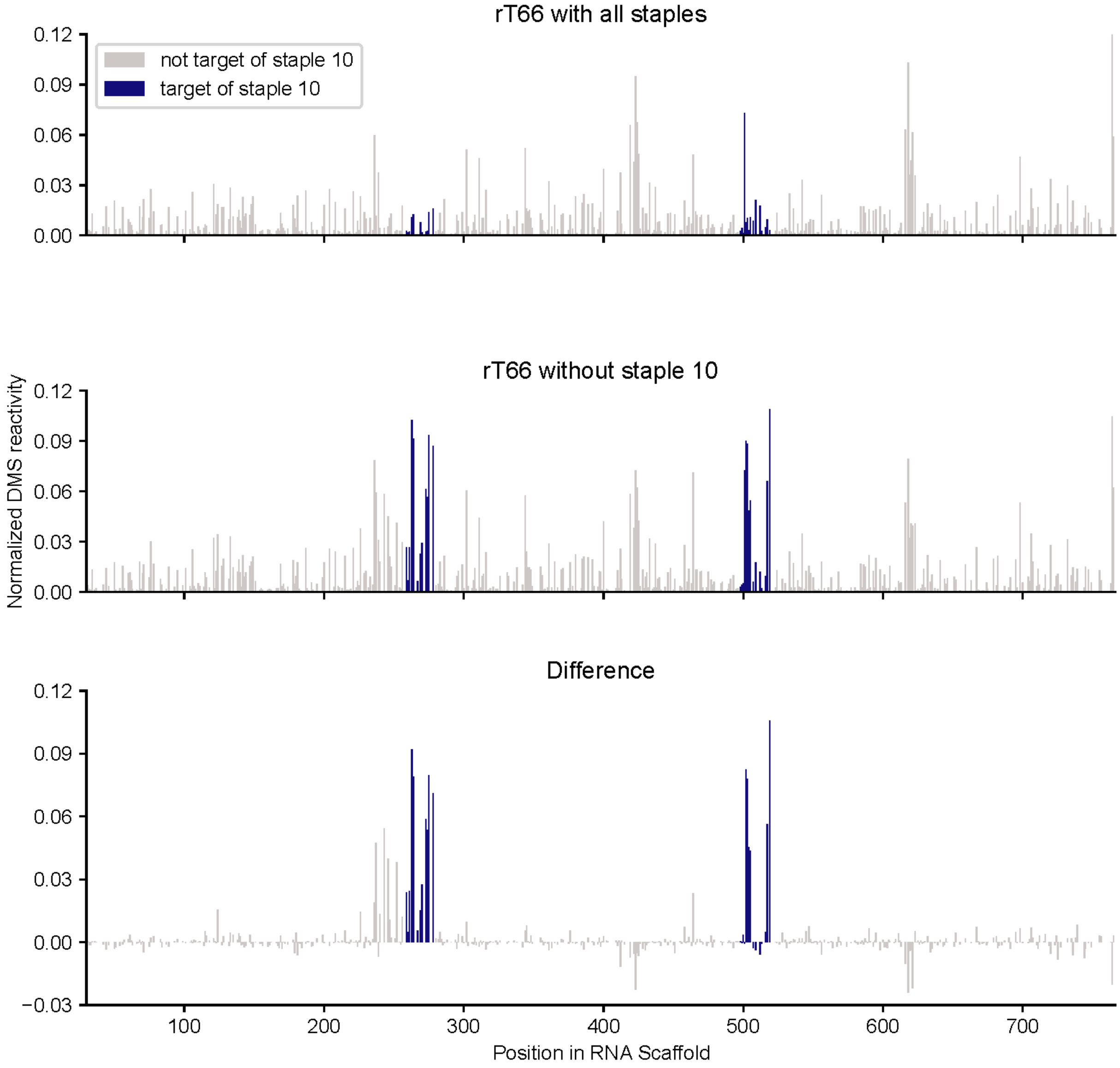

Supplementary Figure 3. **Detection of unhybridized scaffold regions with DMS-MaPseq.** Reactivity profiles for the A-form rT66 scaffolded with EGFP mRNA, folded with (*n* = 1 replicate) and without (*n* = 1 replicate) staple 10. Scaffold nucleotides targeted by staple 10 are colored blue. "Difference" is the DMS reactivity without staple 10 minus the reactivity with staple 10. Source data are provided as a Source Data file.

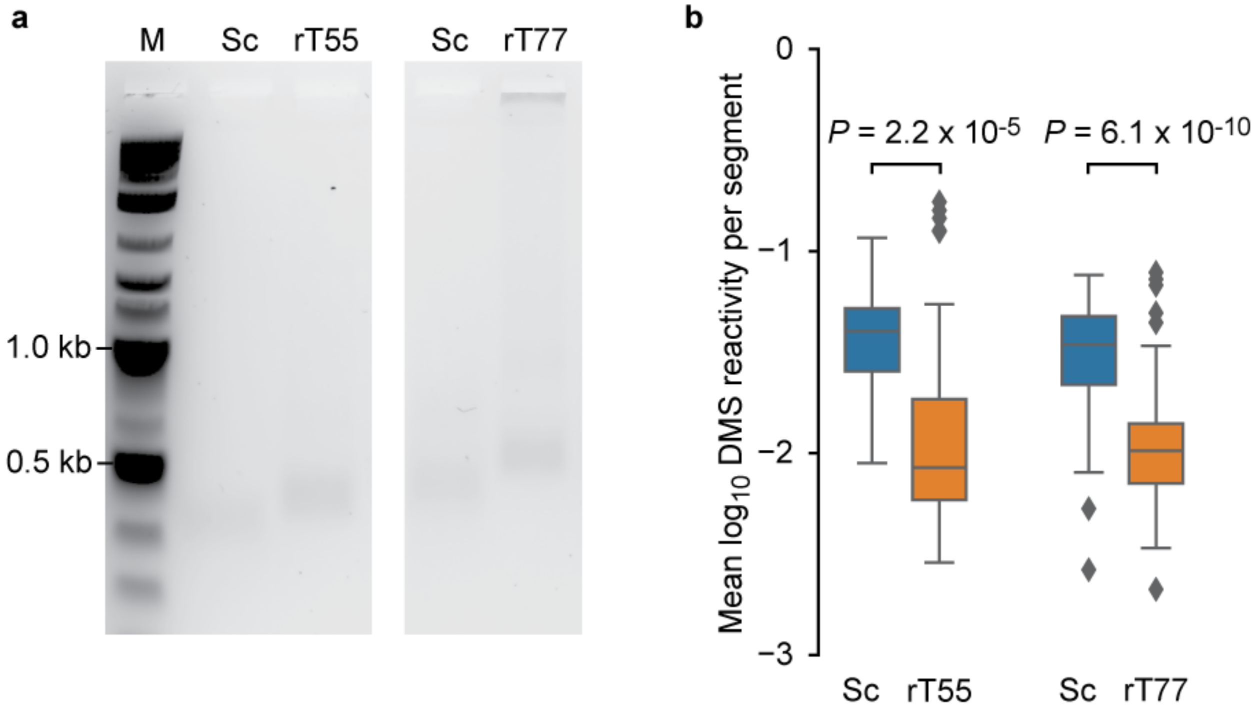

Supplementary Figure 4. **Gel mobility shift assays for A-form RNA-scaffolded tetrahedron with odd edge lengths. a** (left) Tetrahedron with five helical turns per edge, with 'rsc1218v1_T55' synthetic RNA fragment scaffold and (right) tetrahedron with seven helical turns per edge, with 'rsc1218v1_T77' synthetic RNA fragment scaffold, n = 1 replicate each. M indicates the 1 kb plus DNA ladder (NEB) used as a marker. Sc indicates scaffold without DNA staples. **b** Distributions of mean DMS reactivities of adenines and cytosines among RNA:DNA duplex segments in rT55 (n = 53 segments) and rT77 (n = 75 segments) (orange box plots), and among the corresponding bases in their scaffolds folded without staples (Sc, blue box plots), n = 1 replicate each. Each box plot depicts the median (middle line), 1st and 3rd quartiles (box), minimum/maximum up to 1.5 interquartile ranges from box (whiskers), and outliers (grey diamonds). Each P-value indicates the significance of the difference between the left and right distributions (two-sided Wilcoxon signed-rank test). Source data are provided as a Source Data file.

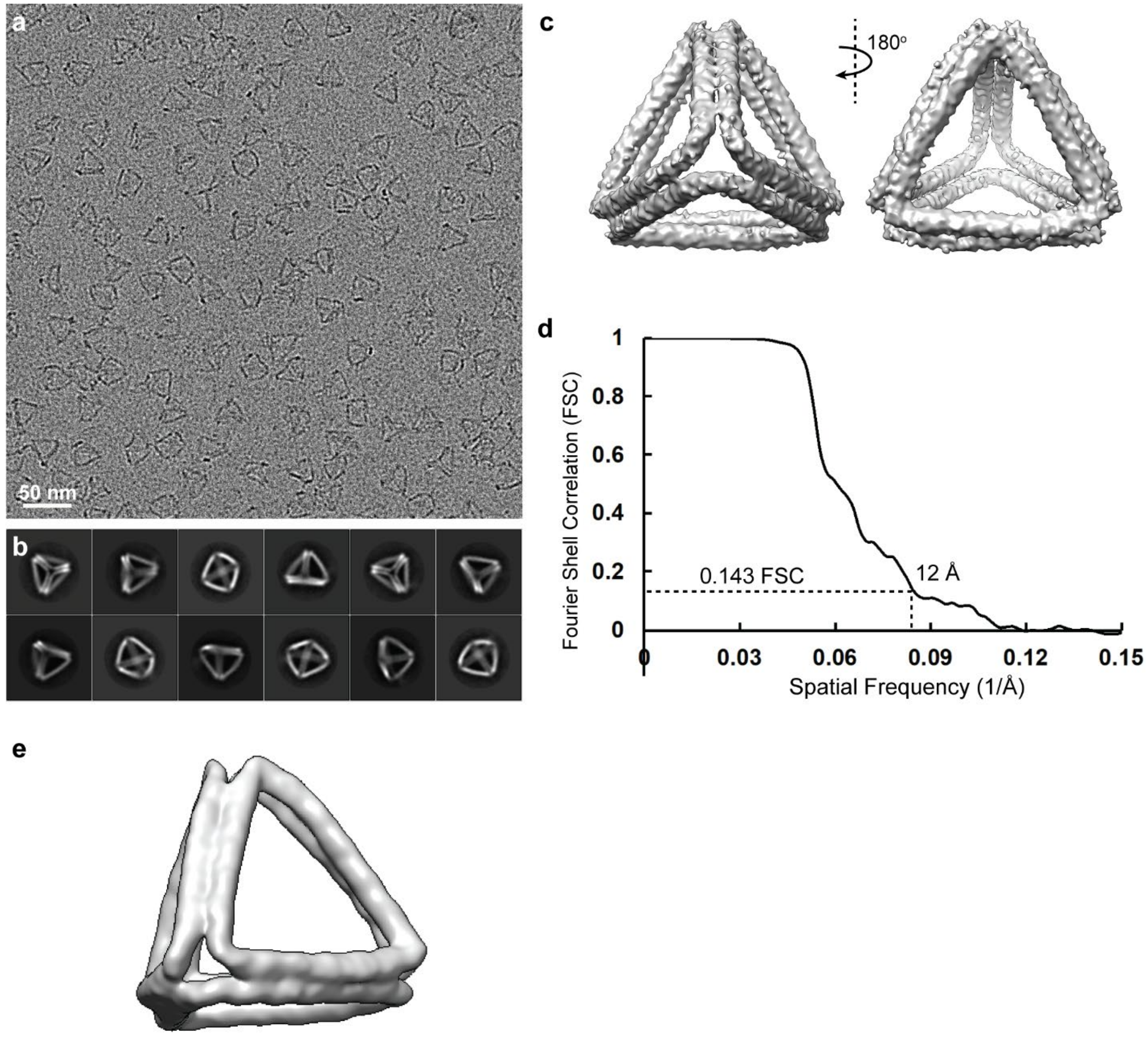

Supplementary Figure 5. **Cryo-EM reconstruction for the A-form tetrahedron with EGFP mRNA scaffold (rT66). a** Representative micrograph, *n* = 1 replicate. **b** 2D class averages. **c** Two views of the reconstruction. **d** Fourier shell correlation plot; the resolution of the reconstruction is 12Å. **e** Reconstruction performed without symmetry.

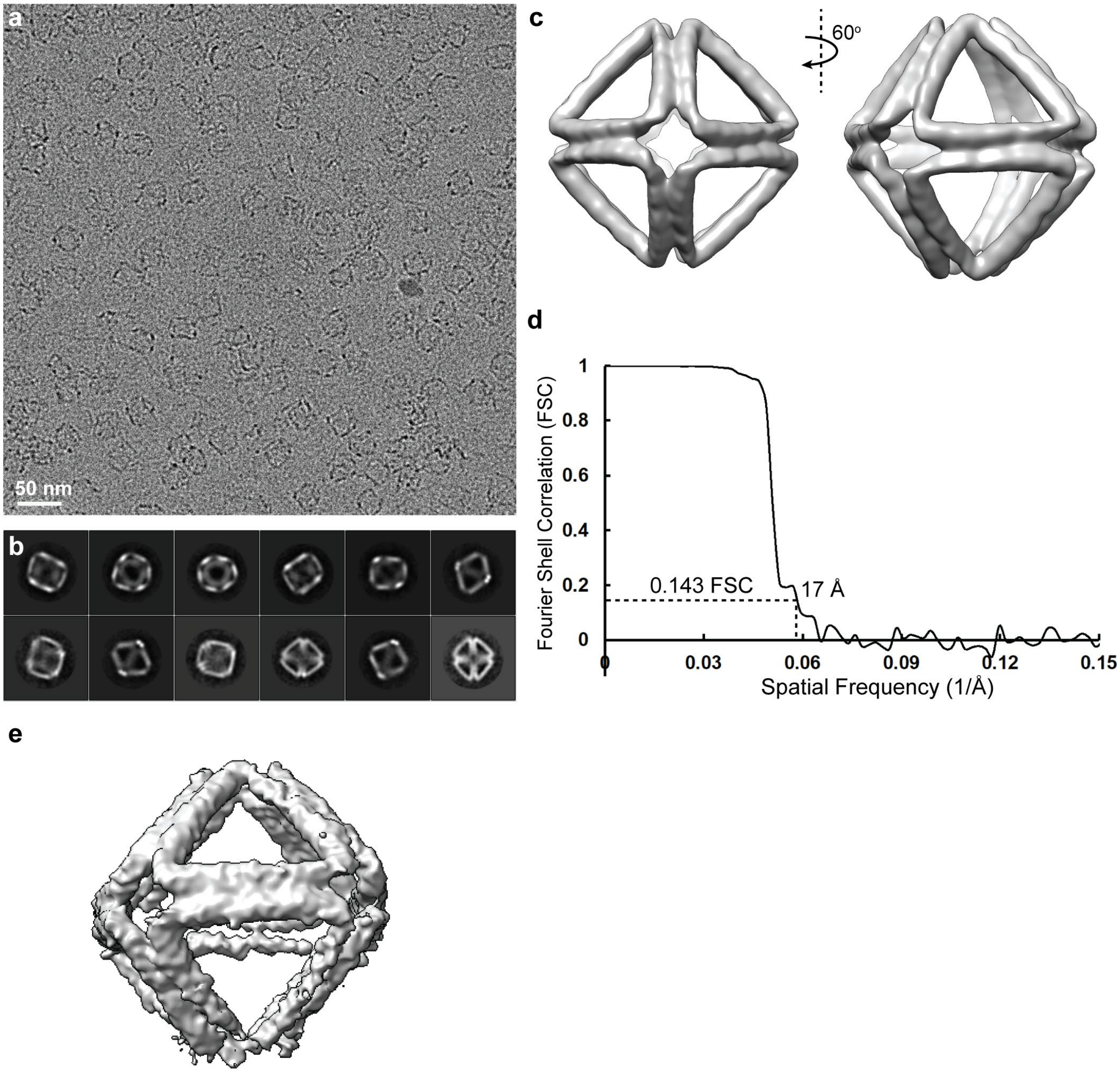

Supplementary Figure 6. **Cryo-EM reconstruction for the A-form octahedron with four helical turns per edge with M13 transcript scaffold (rO44). a** Representative micrograph, $n = 1$ replicate. **b** 2D class averages. **c** Two views of the reconstruction. **d** Fourier shell correlation plot; the resolution of the reconstruction is 17Å. **e** Reconstruction performed without symmetry.

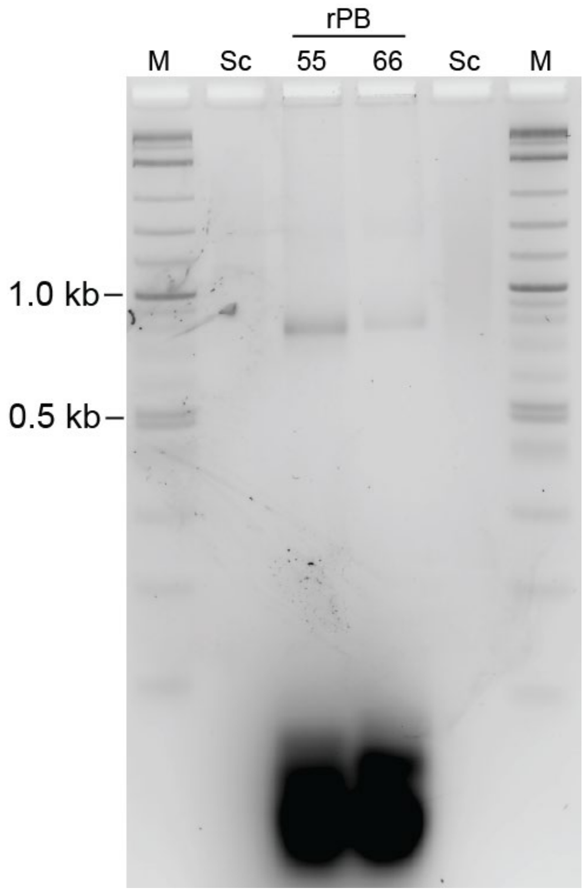

Supplementary Figure 7. **Gel mobility shift assay for A-form pentagonal bipyramid with five helical turns per edge (rPB55), with 23s rRNA fragment scaffold.** This scaffold often forms a smear in agarose gels with salt, likely due to the formation of secondary structure, but it shows a single band on denaturing PAGE gels. The pentagonal bipyramid with six helical turns per edge (rPB66) folded with the same scaffold is included in the gel for comparison, *n* = 1 replicate.

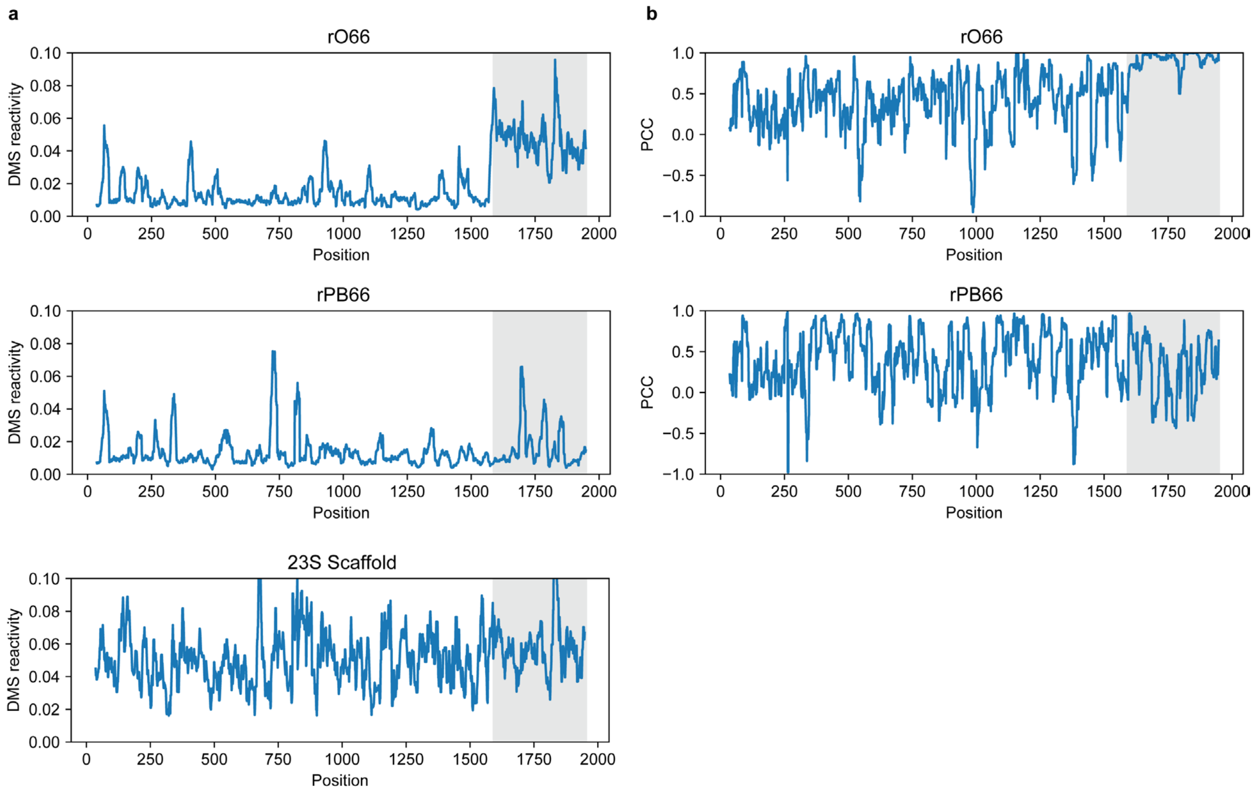

Supplementary Figure 8. **DMS-MaPseq reactivity profiles for A-form 23S-rRNA-scaffolded origami. a** Normalized DMS reactivity for each nucleotide of the scaffold, when incubated with staples to fold the rO66 or the rPB66, or when incubated without staples (bottom), *n* = 1 replicate each. The final 396 nt of the scaffold (highlighted in grey) were not targeted by staples in the rO66. **b** Pearson Correlation Coefficient (PCC) values for the DMS reactivities of the scaffold when folded with staples vs. the scaffold folded alone. The final 396 nt of the scaffold (highlighted in grey) were not targeted by staples in the rO66. Source data are provided as a Source Data file.

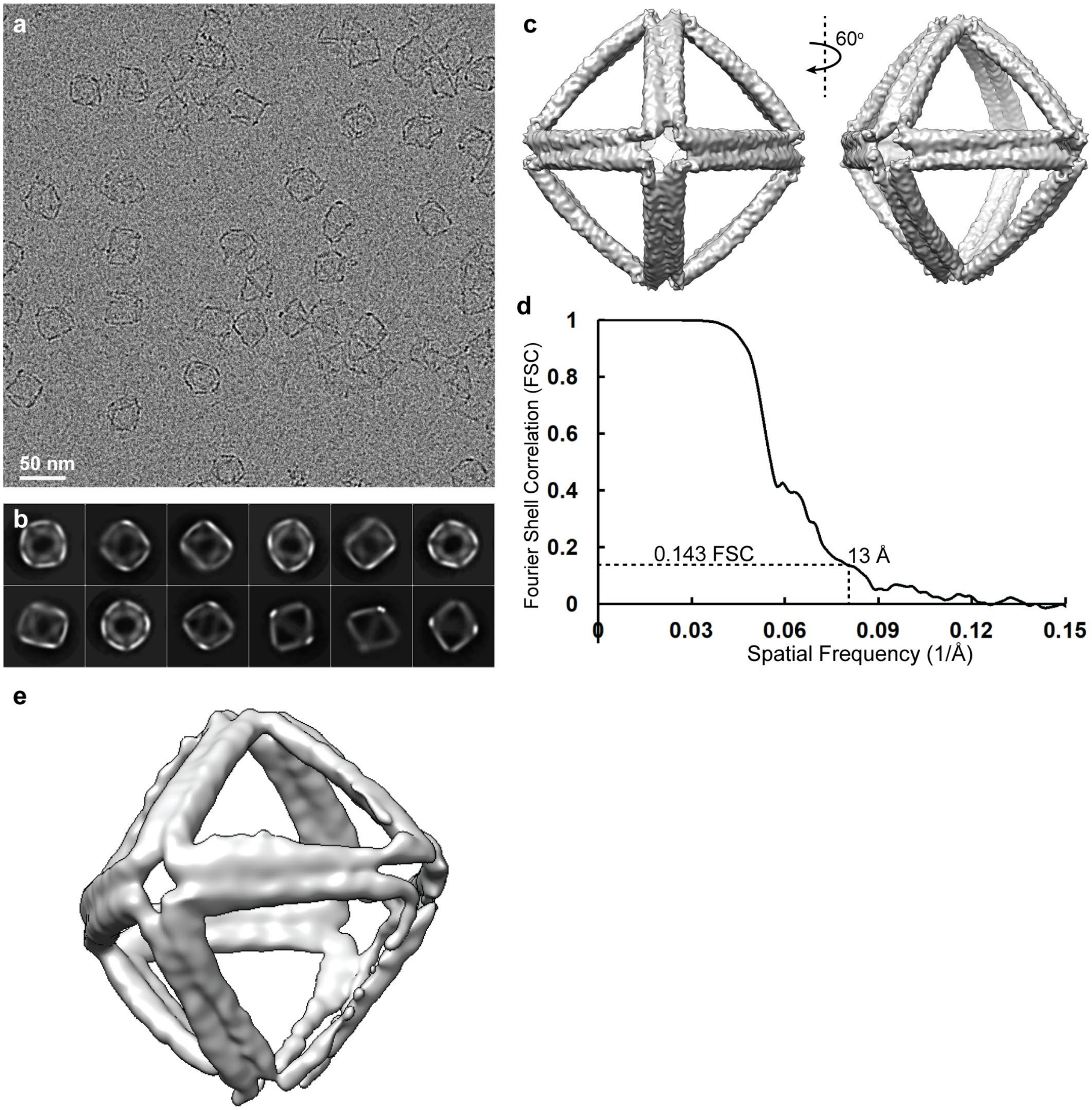

Supplementary Figure 9. **Cryo-EM micrographs for the A-form octahedron with six helical turns per edge and 23s rRNA fragment scaffold (rO66). a** Representative micrograph, $n = 1$ replicate. **b** 2D class averages. **c** Two views of the reconstruction. **d** Fourier shell correlation plot; the resolution of the reconstruction is 13Å. **e** Reconstruction performed without symmetry.

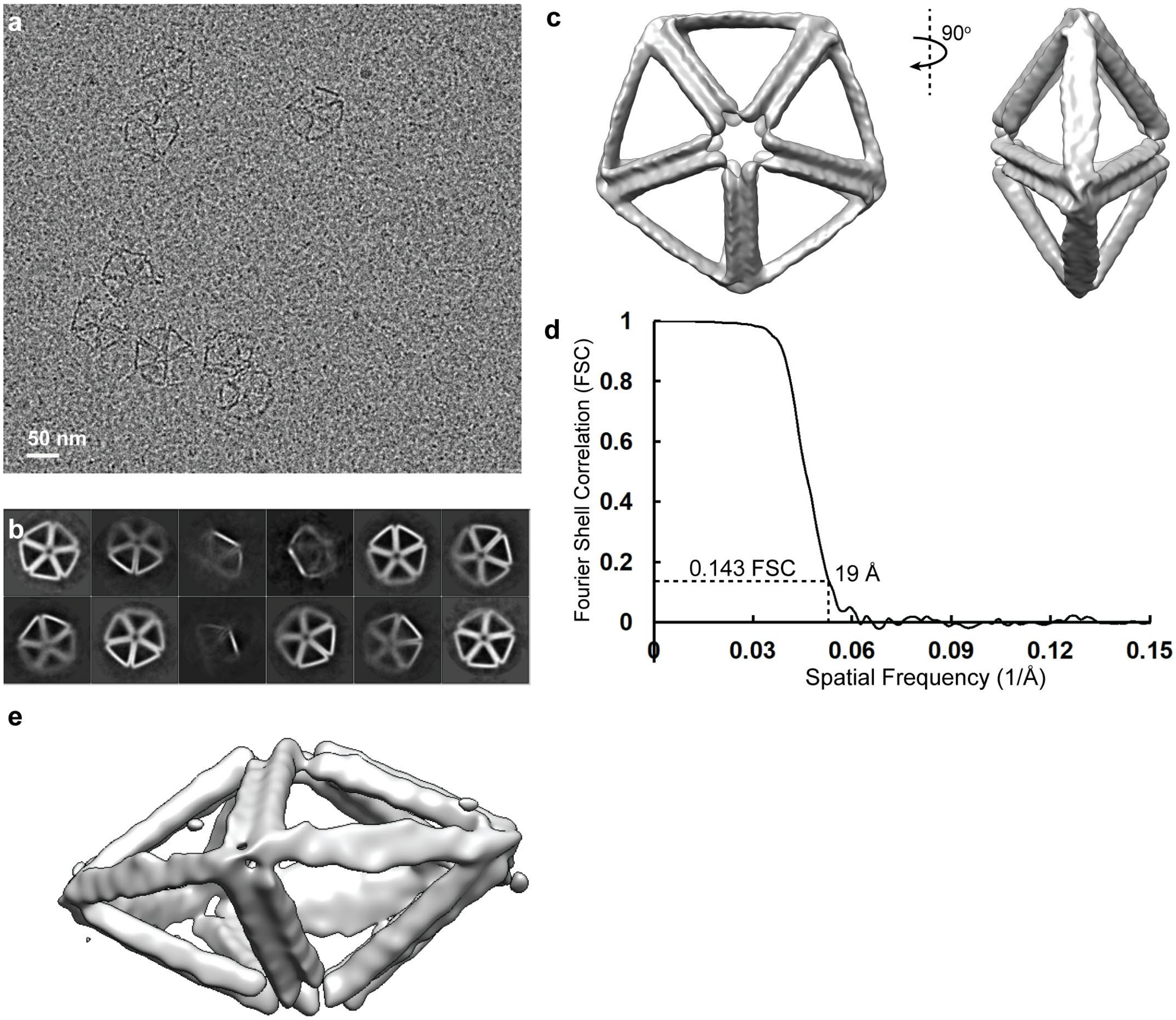

Supplementary Figure 10. **Cryo-EM reconstruction for the A-form pentagonal bipyramid with 23s rRNA fragment scaffold (rPB66). a** Representative micrograph, *n* = 1 replicate. **b** 2D class averages. **c** Two views of the reconstruction. **d** Fourier shell correlation plot; the resolution of the reconstruction is 19Å. **e** Reconstruction performed without symmetry.

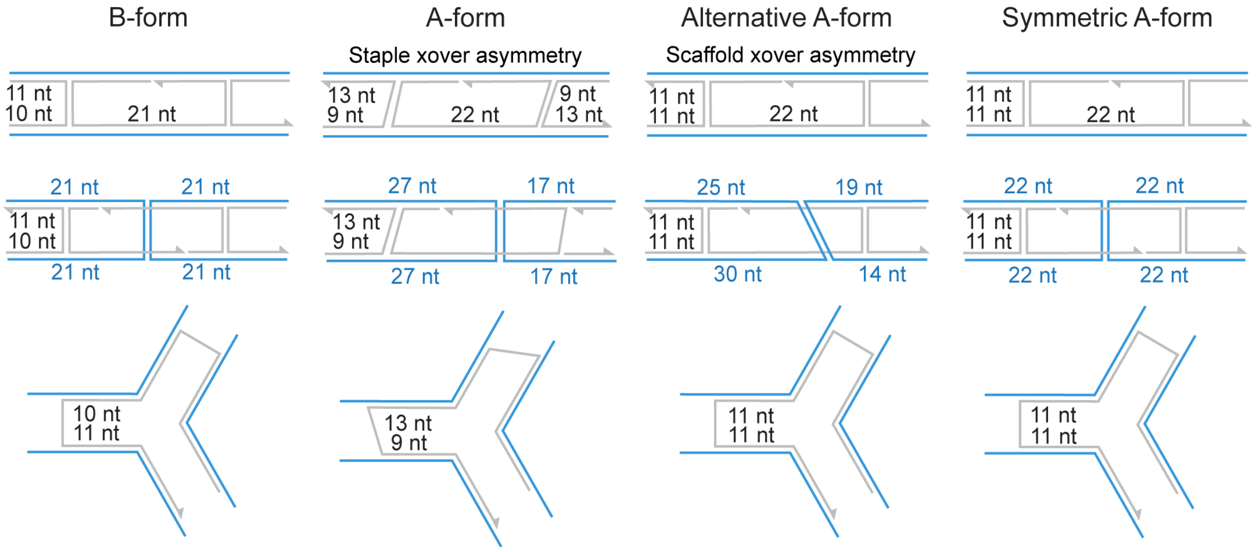

Supplementary Figure 11. **Comparison of B-form (DAEDALUS[1]), A-form, Alternative A-form (Alt A-form), and Symmetric A-form (Sym A-form) scaffold and staple edge and vertex routings.** “xover” denotes “crossover.”

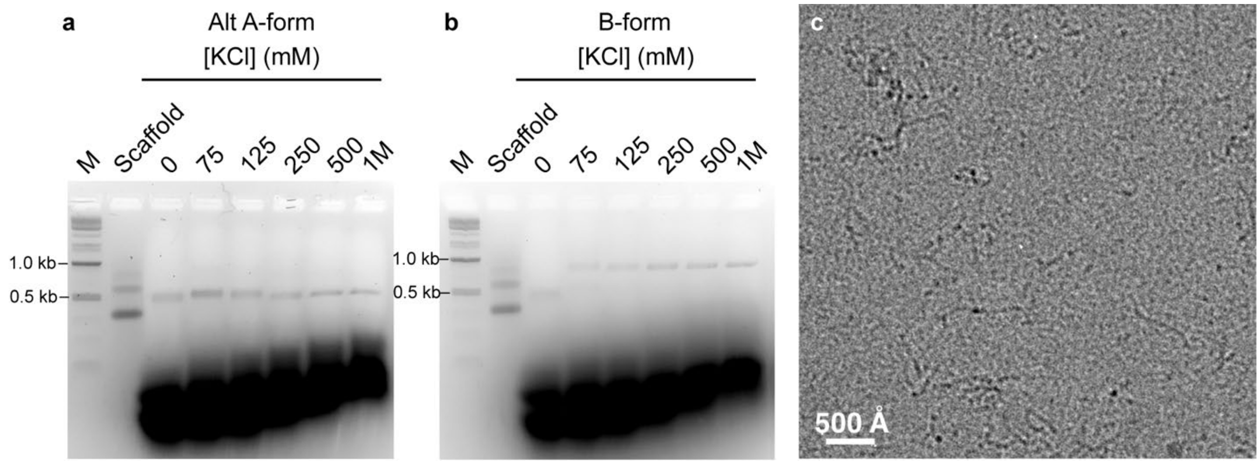

Supplementary Figure 12. **B-form staple designs from DAEDALUS[1] used to fold EGFP mRNA-scaffolded origami. a** KCl titration of Alt A-form staple designs with RNA scaffolding, designed to fold a tetrahedron with six helical turns (66 bp) per edge, *n* = 1 replicate. **b** KCl titration of B-form staple designs with RNA scaffolding, designed to fold a tetrahedron with six helical turns (63 bp) per edge, with the band being noticeably higher, suggesting a less compact (less complete) folded product, *n* = 1 replicate. **c** Cryo-electron microscopy micrograph showing the unfolded structure of the B-form-stapled origami, *n* = 1 replicate.

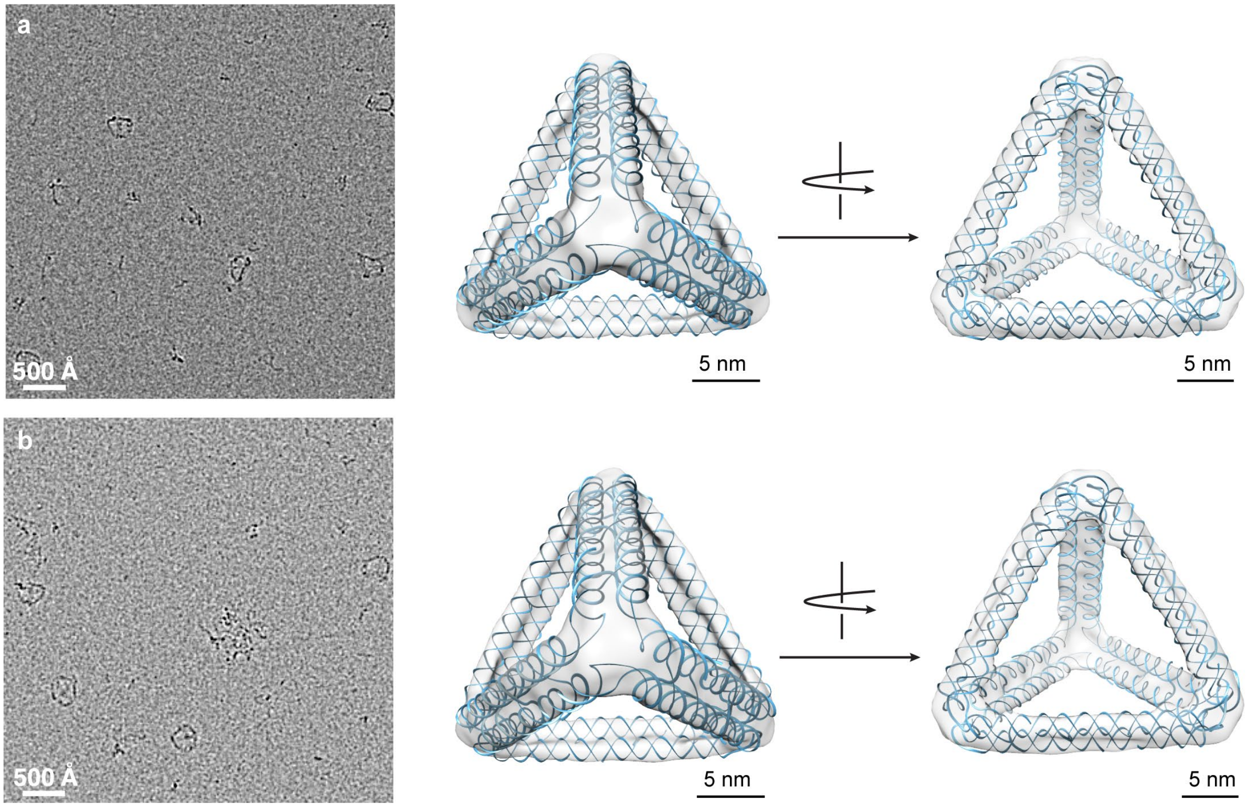

Supplementary Figure 13. **Cryo-EM comparison of EGFP mRNA-scaffolded tetrahedron with 66-bp edge lengths.** Representative micrograph and reconstruction fit to model for the tetrahedron folded using **a** Alt A-form geometry staples (*n* = 1 replicate), or **b** Sym A-form geometry staples (*n* = 1 replicate).

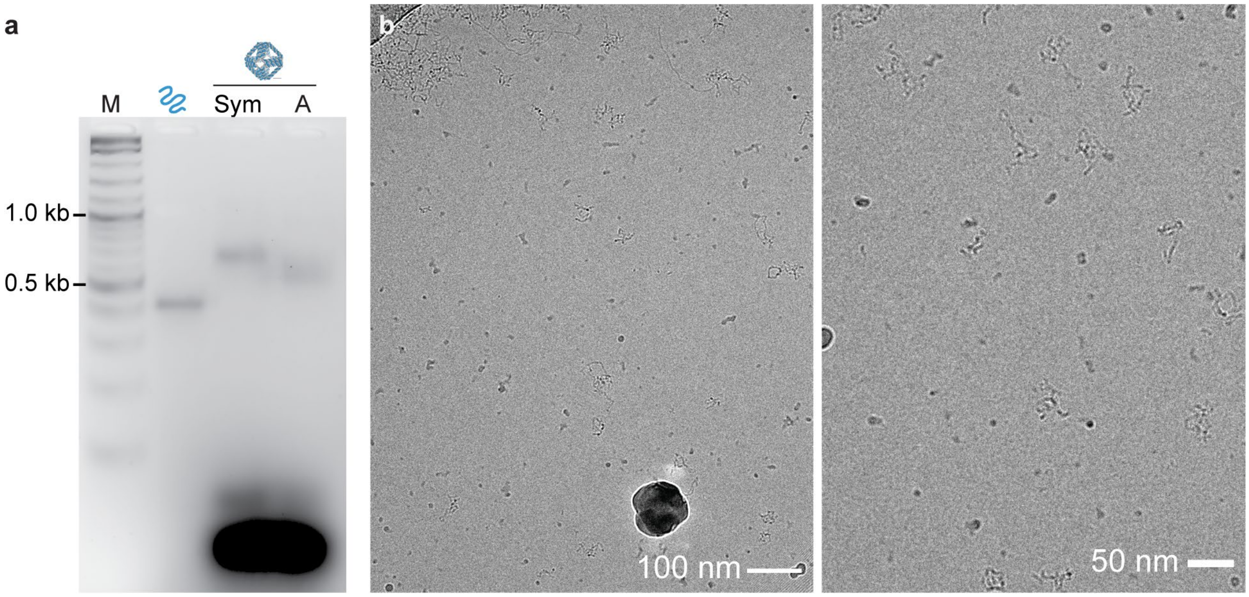

Supplementary Figure 14. **Cryo-EM micrographs for a Sym A-form regular octahedron with four helical turns (44 bp) per edge, scaffolded with M13 transcript RNA. a** Gel mobility shift assay comparing the Sym A-form and A-form octahedra folds to the scaffold without staples, *n* = 1 replicate. The Sym A-form fold leads to a notably higher band shift than the A-form fold. The marker (M) is 1 kb plus DNA ladder from NEB. **b** Cryo-EM micrographs of the Sym A-form octahedron, showing no well-folded octahedral particles, *n* = 1 replicate.

Supplementary Note: For an alternative A-form (Alt A-form) EGFP mRNA-scaffolded tetrahedron with six helical turns (66 bp) per edge, gel mobility shift assays after folding with KCl or NaCl showed an upward shift of the major band relative to unpaired scaffold, with the band position and breadth stabilized at 300 mM monovalent salt (Supplementary Fig. 15). These results suggested RNA:DNA origami wireframe particles were properly folded in 300 mM monovalent salt and 10 mM HEPES-KOH pH 7.5. No major band was observed when attempting to fold the tetrahedron in magnesium, as expected, likely due to RNA degradation from this divalent salt at elevated temperatures during annealing (Supplementary Fig. 16). Consistent with this hypothesis, this effect was mitigated by using a fast-folding protocol[2] (Supplementary Fig. 17). Higher yields of the folded particles were achieved in HEPES-KOH pH 7.5 buffer than in Tris-HCl pH 8.1 buffer (Supplementary Fig. 17), possibly due to the combined effects of higher pH and temperature.

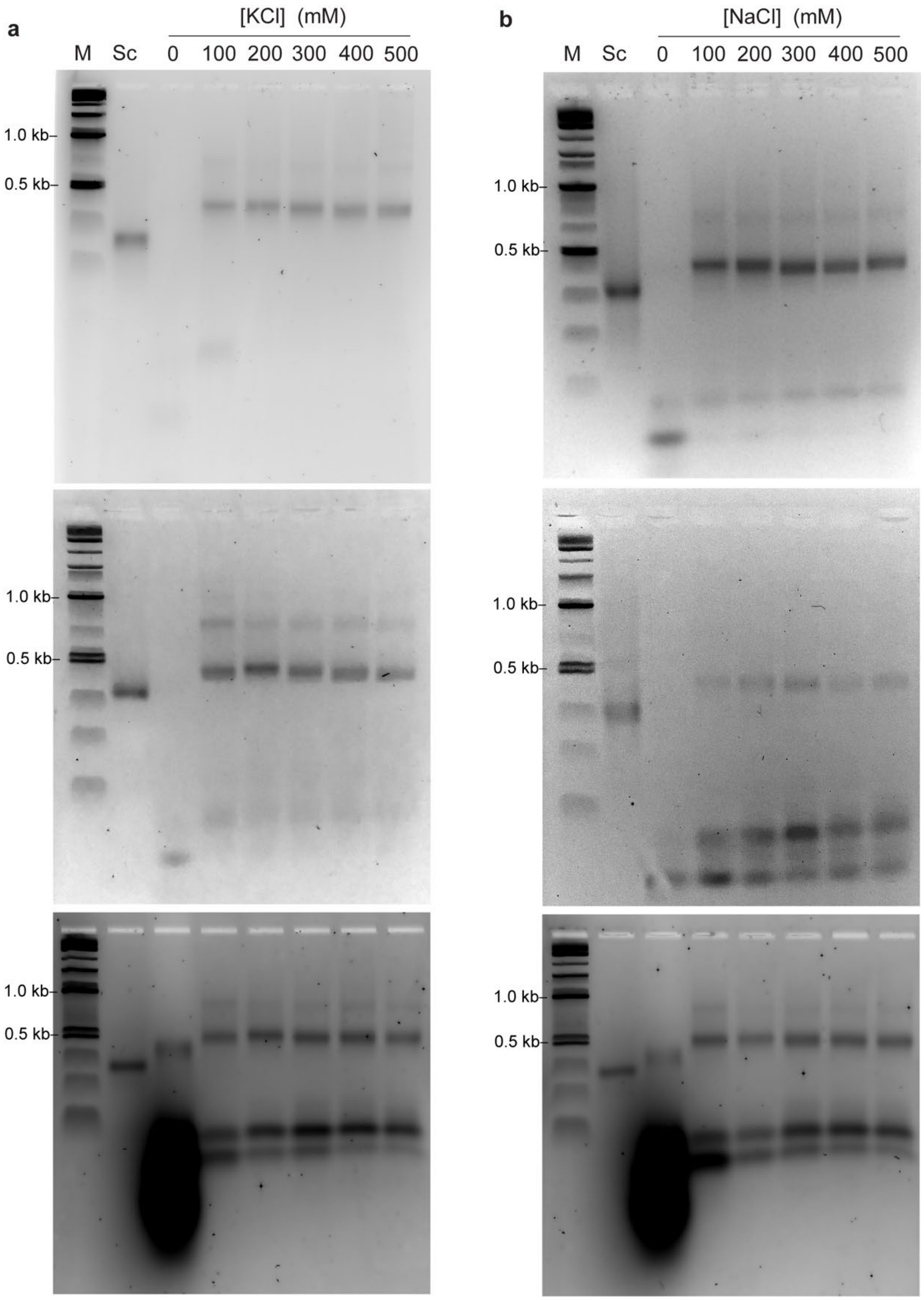

Supplementary Figure 15. **Titration of folding in monovalent salts and 10 mM HEPES-KOH pH 7.5 for the Alt A-form rT66. a** Titration series of KCl, n = 3 replicates. **b** Titration series of NaCl, n = 3 replicates. M is 1 kb plus DNA ladder from New England Biosciences. Sc indicates the scaffold alone. The folded origami bands shift slightly up relative to the scaffold band, with mobility and breadth stabilizing at 300 mM salt.

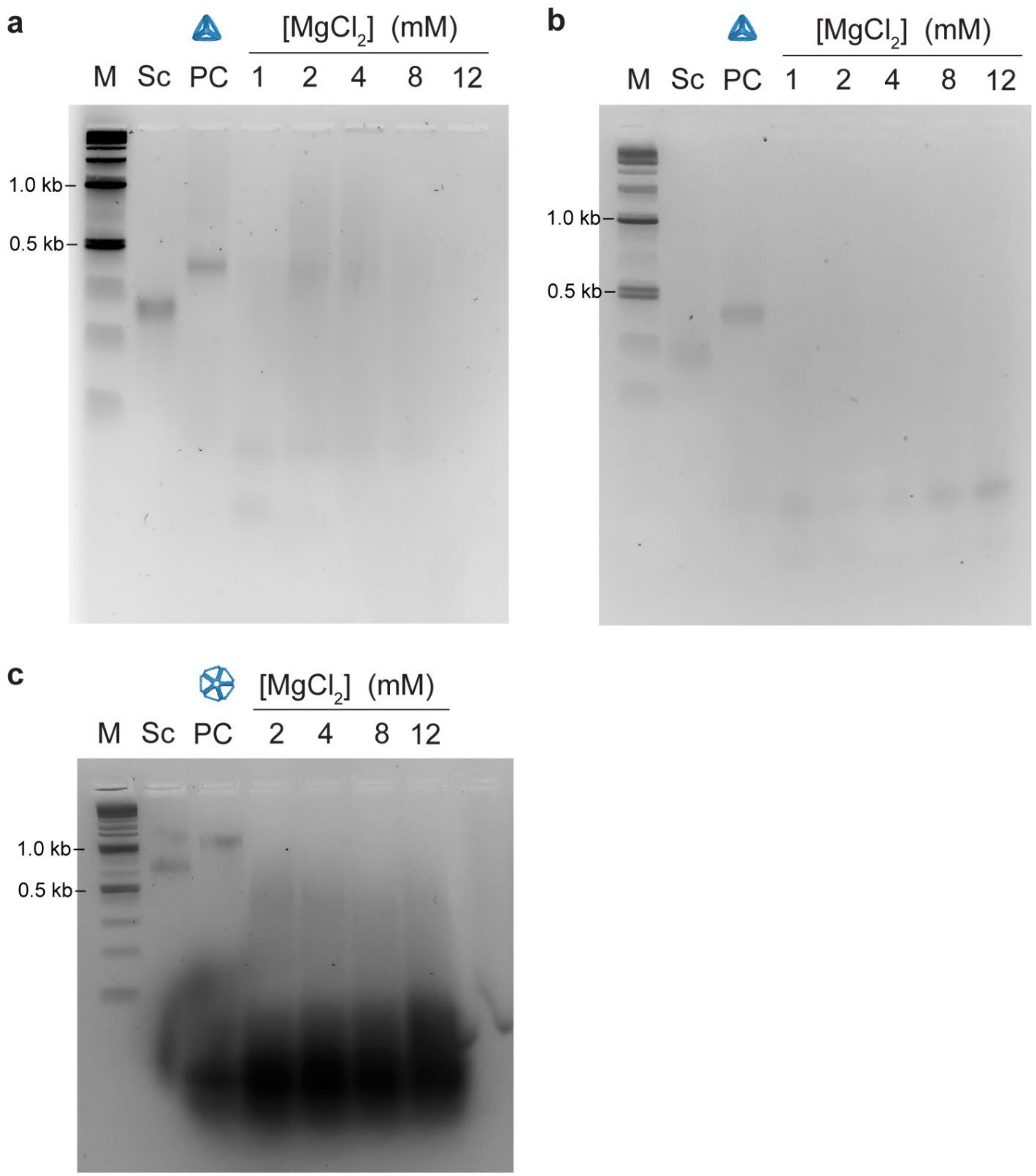

Supplementary Figure 16. **Magnesium titration for folding Alt A-form objects. a** and **b** Titrations of folding in $MgCl_2$ and 10 mM HEPES-KOH pH 7.5 for the Alt A-form rT66, *n* = 2 replicates. **c** A titration of folding in $MgCl_2$ for the Alt A-form rPB66, *n* = 1 replicate. M is 1 kb plus DNA ladder from New England Biosciences. Sc indicates the scaffold alone. PC indicates a positive control of the origami folded in 300 mM KCl and 10 mM HEPES-KOH pH 7.5. In all cases, no discrete band corresponding to the folded origami or scaffold appears at any concentration of magnesium, suggesting at least partial degradation of the RNA scaffold.

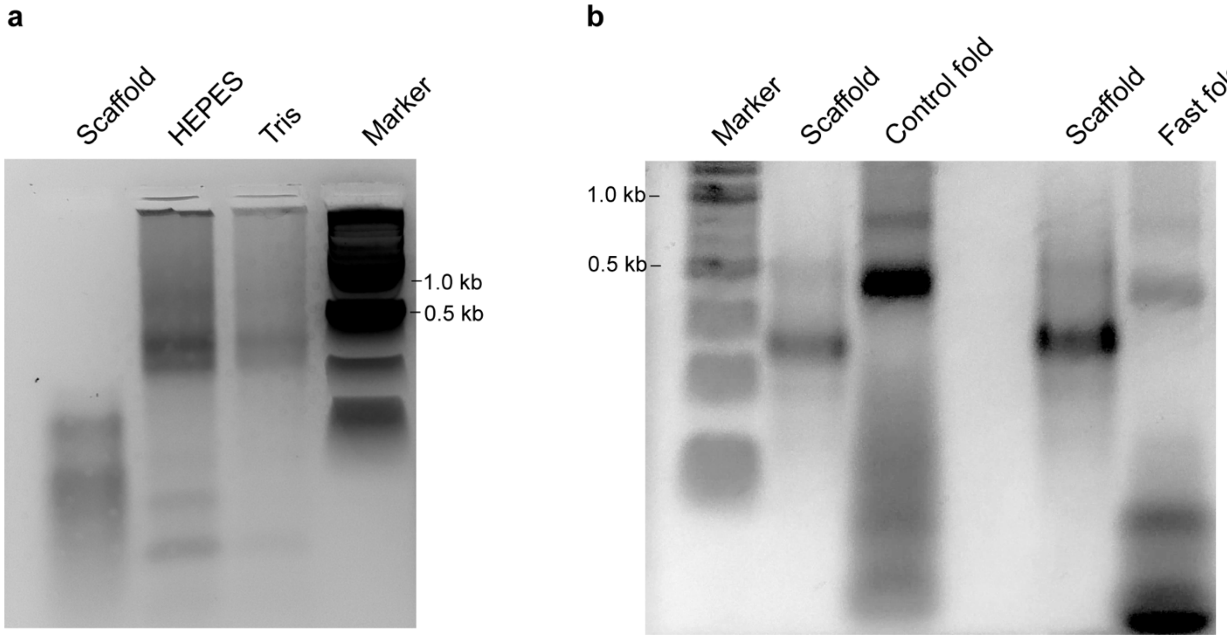

Supplementary Figure 17. **Comparison of alternative folding conditions for the Alt A-form rT66. a** HEPES pH 7.5 was compared to Tris-HCl pH 8.1 for the 13-h folding protocol modified from DAEDALUS[1], both with KCl, *n* = 1 replicate. **b** HEPES pH 7.5 and KCl with the 13-h folding protocol ("Control fold") was tested against a previously published 40-min protocol in TAE buffer with magnesium ("Fast fold"[2]), showing nearly equivalent yields when adjusted for loading amounts, *n* = 1 replicate.

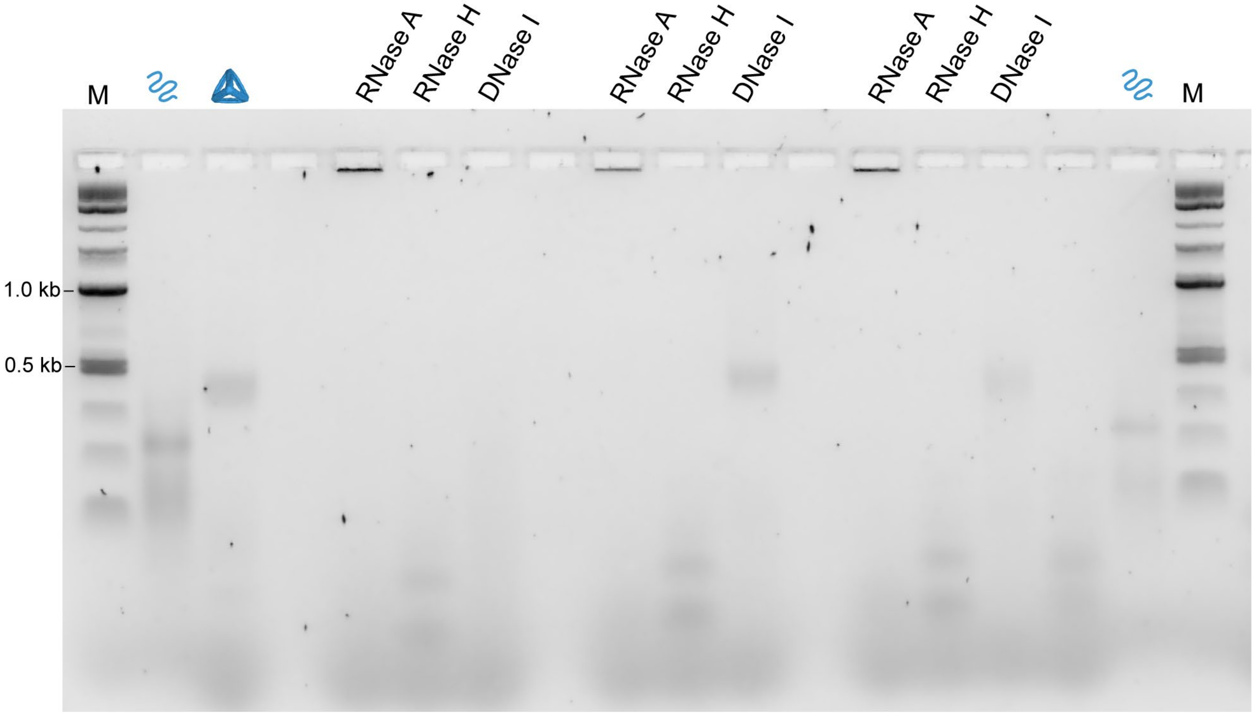

Supplementary Figure 18. **Characterization of biochemical stability of the Alt A-form EGFP mRNA-scaffolded tetrahedron with 66-bp edge length, using canonical nucleotides.** The RNase A-, Rnase H-, and Dnase I-labeled lanes all represent the folded rT66 treated with the respective nuclease for 5 min at 37ºC, n = 3 replicates each. Two of the three Dnase I replicates show the intact folded origami band. Rnase H, which specifically targets RNA hybridized to DNA, degraded the folded origami and released staples within five minutes, with the folded origami band completely disappearing, the scaffold strand appearing to be fully digested, and bands corresponding to DNA staples concomitantly appearing. This degradation additionally confirmed trace template DNA was not responsible for forming the principal origami product. With Rnase A treatment, no folded origami gel band showed after five minutes incubation, and some aggregation appeared, possibly due to intact origami-bound Rnase A without cleavage because of the steric hindrance in the double-duplex edges. This interpretation is supported by the absence of discrete bands corresponding to DNA staple strands after Rnase A treatment, since they are not digested by Rnase A. Interestingly, Dnase I did not appear to degrade incorporated DNA staples within a 5-minute incubation, suggesting that nanostructuring with RNA might offer some protection to DNA staples from enzymatic degradation.

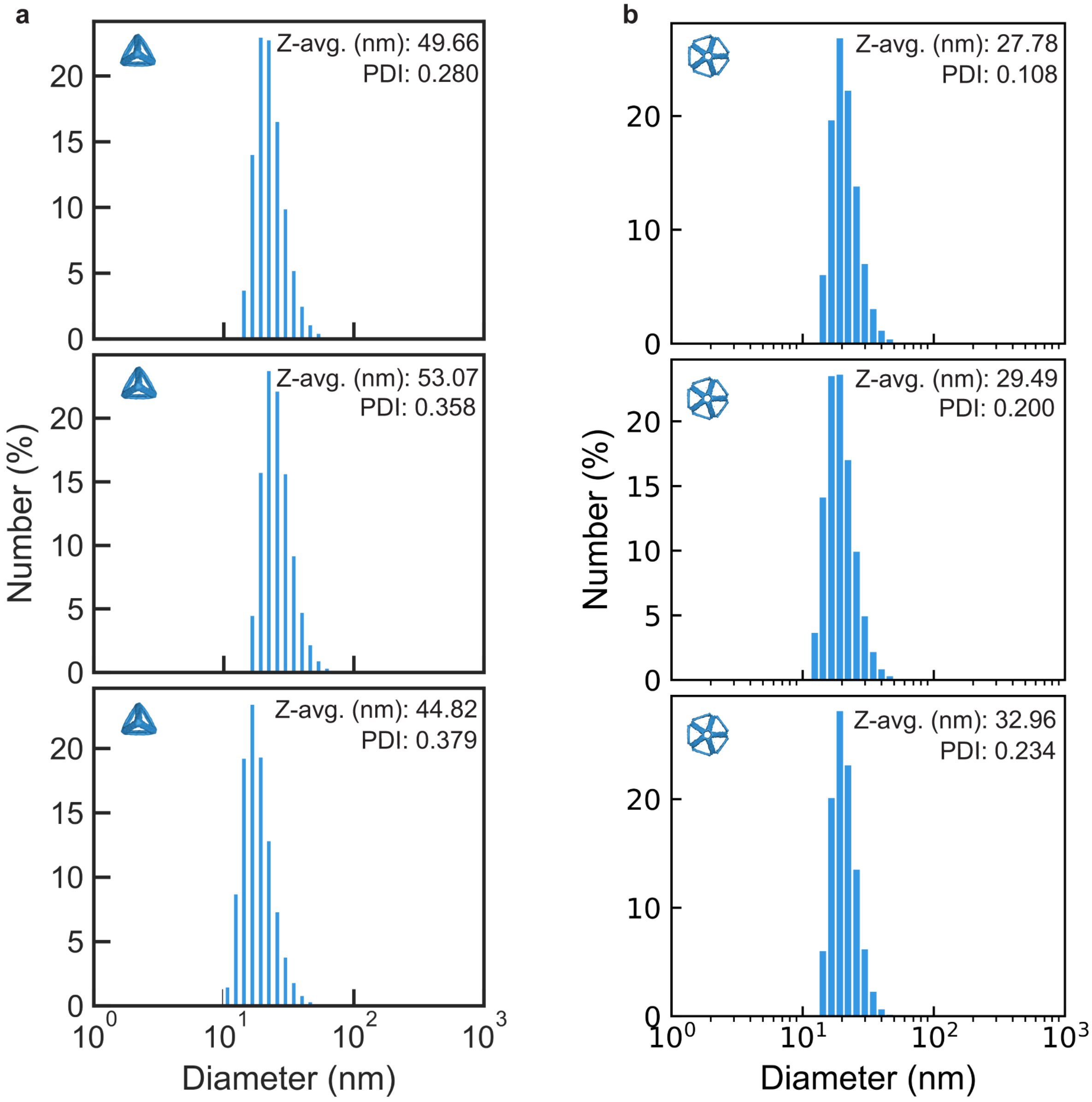

Supplementary Figure 19. **Dynamic light scattering (DLS) showing monodisperse objects. a** Alt A-form rT66, *n* = 3 replicates. The size (hydrodynamic diameter) at the peak of the distribution by number is 17.86 nm ± 5.67 nm. The designed edge length is 17.16 nm and expected tetrahedral height is approximately 14 nm. **b** Alt A-form rPB66, *n* = 3 replicates. The size (hydrodynamic diameter) at the peak of the distribution by number is 20.38 nm ± 5.10 nm. The designed edge length is 17.16 nm and expected height and width of the pentagonal bipyramid are approximately 21 nm x 36 nm. Source data are provided as a Source Data file.

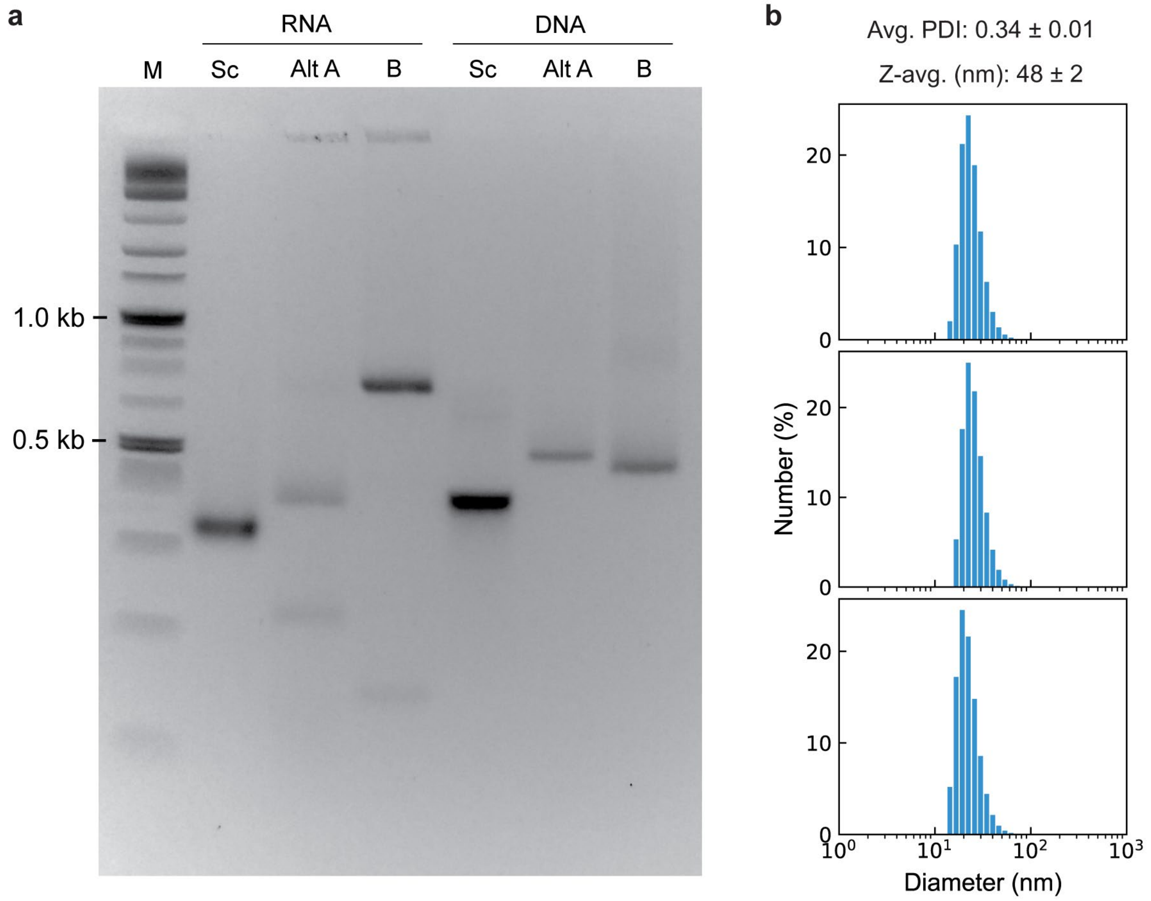

Supplementary Figure 20. **Comparison of RNA- and DNA-scaffolded tetrahedra with six helical turns (66 bp for Alt A-form, 63 bp for B-form) per edge. a** Prokaryotic EGFP mRNA sequence was used for either RNA or DNA scaffold (Sc), folded with DNA staples designed for Alt A-form in 10 mM HEPES and 300 mM KCl; or folded with DNA staples designed for B-form routing following the DAEDALUS protocol[1], n = 1 replicate each. All folded objects show discrete bands that are shifted slightly upwards relative to the scaffold band, suggesting proper folding, with the exception of RNA scaffold folded with B-form staples that leads to an extreme upward band shift and appears to not have folded compactly. **b** Dynamic light scattering measurement of the B-form DNA-scaffolded tetrahedron with six helical turns (63 bp) per edge, n = 3 replicates. The size (hydrodynamic diameter) at the peak of the distribution by number is 21.46 nm $\pm$ 6.46 nm. The designed edge length is 21.42 nm and expected height is approximately 17 nm. As expected, the DNA-scaffolded B-form tetrahedron had a slightly larger hydrodynamic diameter than the A-form tetrahedron with the same number of helical turns per edge, corresponding to the greater axial rise per duplex turn in B-form compared with A-form duplex geometries. Source data are provided as a Source Data file.

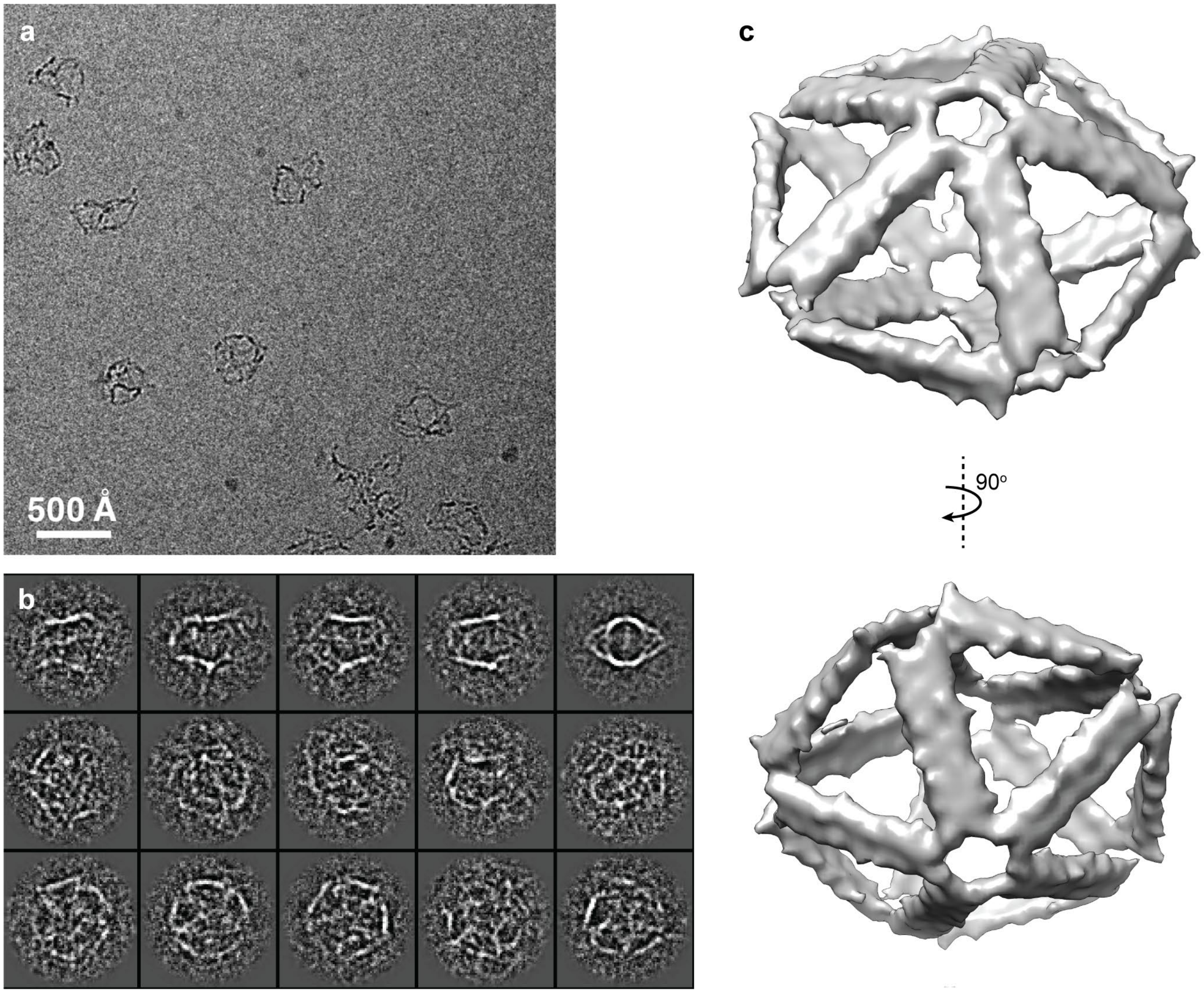

Supplementary Figure 21. **Cryo-EM micrograph of Alt A-form RNA-scaffolded pentagonal bipyramid with 66-bp edge lengths. a** Representative micrograph, *n* = 1 replicate. **b** 2D class averages. **c** Two orthogonal views of the reconstruction.

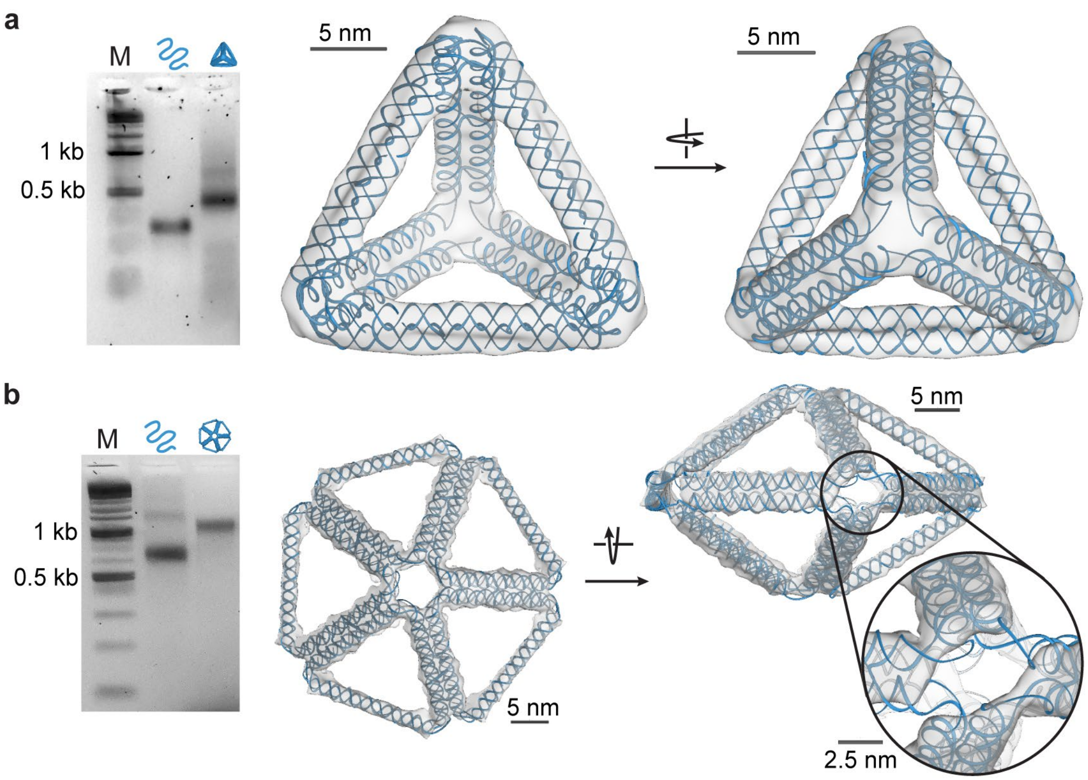

Supplementary Figure 22. **3D structural characterization of Alt A-form RNA-scaffolded wireframe nanoparticles. a** A regular Alt A-form RNA-scaffolded tetrahedron showing the distinct wireframe structure, n = 1 replicate. **b** A regular Alt A-form RNA-scaffolded pentagonal bipyramid with 66-bp edge lengths showing the wireframe structure, n = 1 replicate. A notable twist is seen along the edge, which disrupts the electron density at the vertices, as shown.

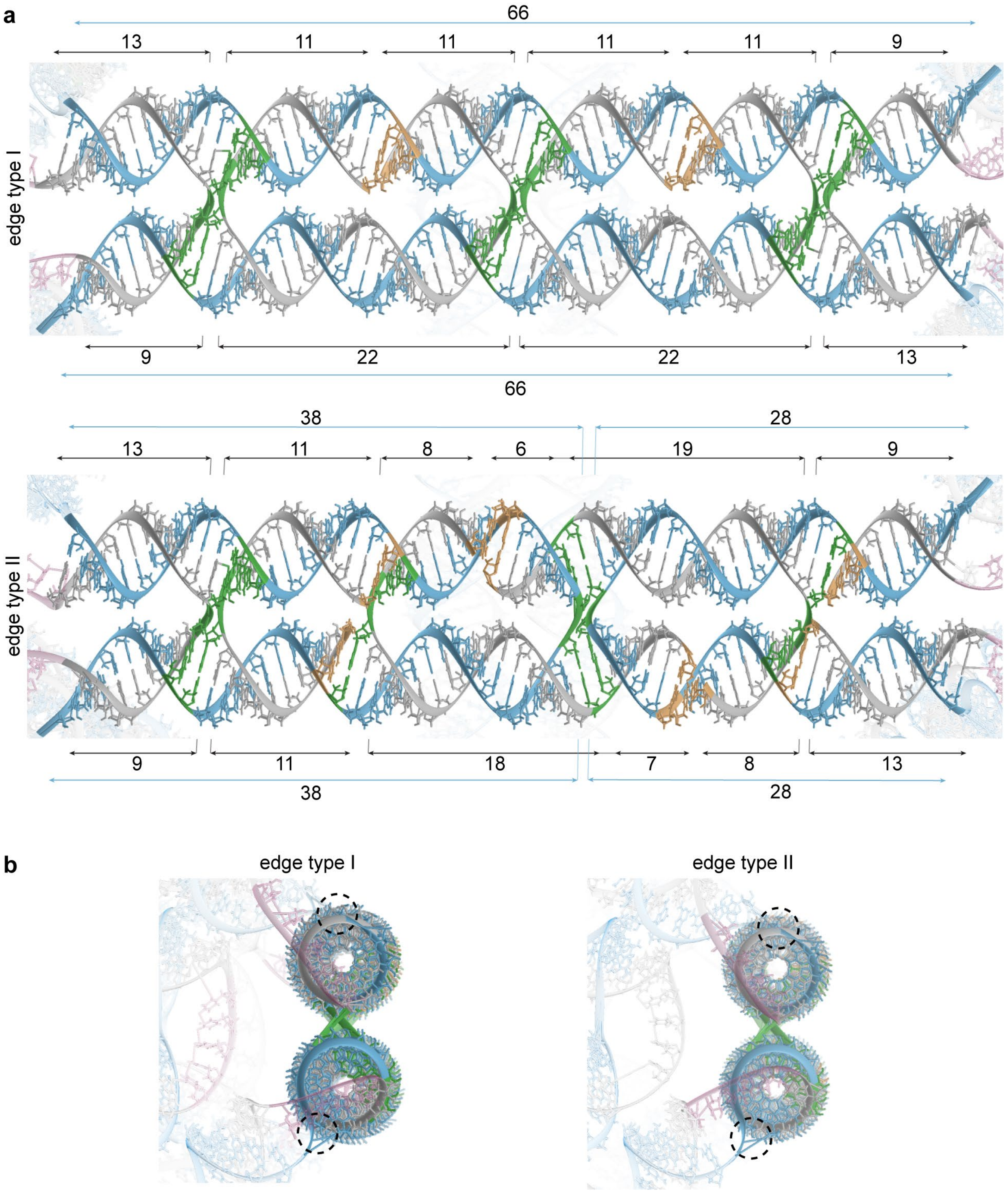

Supplementary Figure 23. **Close-up of edges on the output 3D structure predictions for an A-form tetrahedron with six helical turns per edge, showing both an edge with and an edge without a scaffold crossover. a** Top-down views of the full edges. The connectivity and nucleotide counts in the structure predictions matches the intended design (schematized in Supplementary Figure 11 for four helical turns per edge). Blue: scaffold strand. Grey: staple strands. Green: base pairs at crossovers. Yellow: base pairs at staple nicks. Pink: poly-T loops,

bridging to neighboring edges. **b** End views of the edges. The atomic model is generated with an assumption that the scaffold (blue) will exit the duplex at a helical position opposite the other duplex of the edge, so that the scaffold is near to its entry point in the subsequent edge and no unpaired scaffold is required.

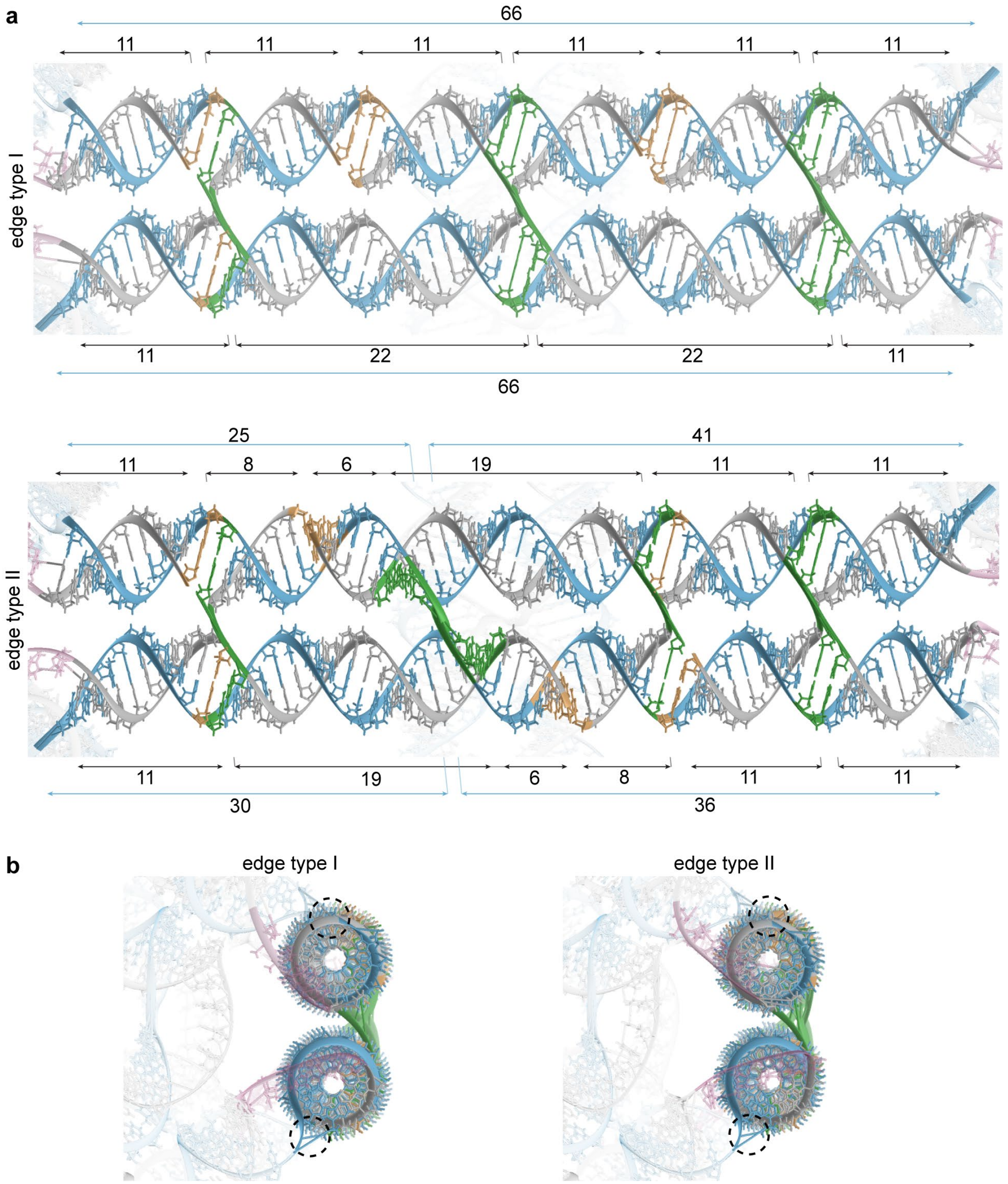

Supplementary Figure 24. **Close-up of edges on the output 3D structure predictions for an Alt A-form tetrahedron with six helical turns per edge, showing both an edge without (type I) and an edge with (type II) a scaffold crossover. a** Top-down view of the full edges. The connectivity and nucleotide counts in the structure predictions matches the intended design (schematized in Supplementary Figure 11 for four helical turns per edge). Because the algorithm generates the structure prediction by affixing the vertex nucleotides for the scaffold on the top and

bottom helices and interpolating nucleotide positions between, there is steric clash at crossovers in the output structure drawing. This clash might be avoided by the scaffold nucleotides entering the vertex from a different point in the helical rotation. Blue: scaffold strand. Grey: staple strands. Green: base pairs at crossovers. Yellow: base pairs at staple nicks. Pink: poly-T loops, bridging to neighboring edges. **b** End views of the edges. The atomic model is generated with an assumption that the scaffold (blue) will exit the duplex at a helical position opposite the other duplex of the edge, so that the scaffold is near to its entry point in the subsequent edge and no unpaired scaffold is required.

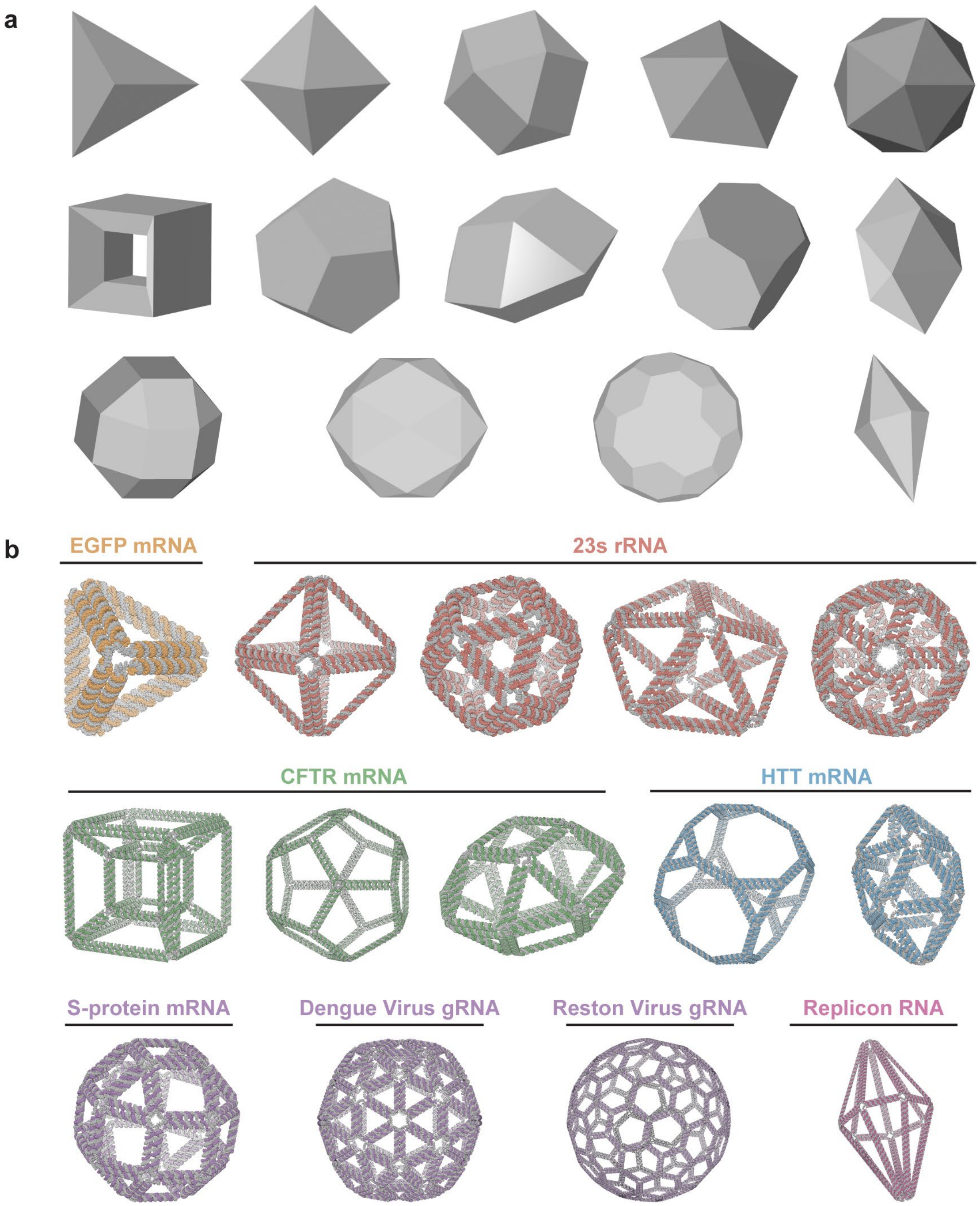

Supplementary Figure 25. **Fourteen A-form objects designed with the pyDAEDALUSX software. a** Input geometries, and **b** output 3D structure predictions, using a variety of RNA scaffold sequence inputs. Geometries and edge lengths were chosen for each sequence such that all or a large fragment of the input scaffold sequence would be incorporated into the folded structure design.

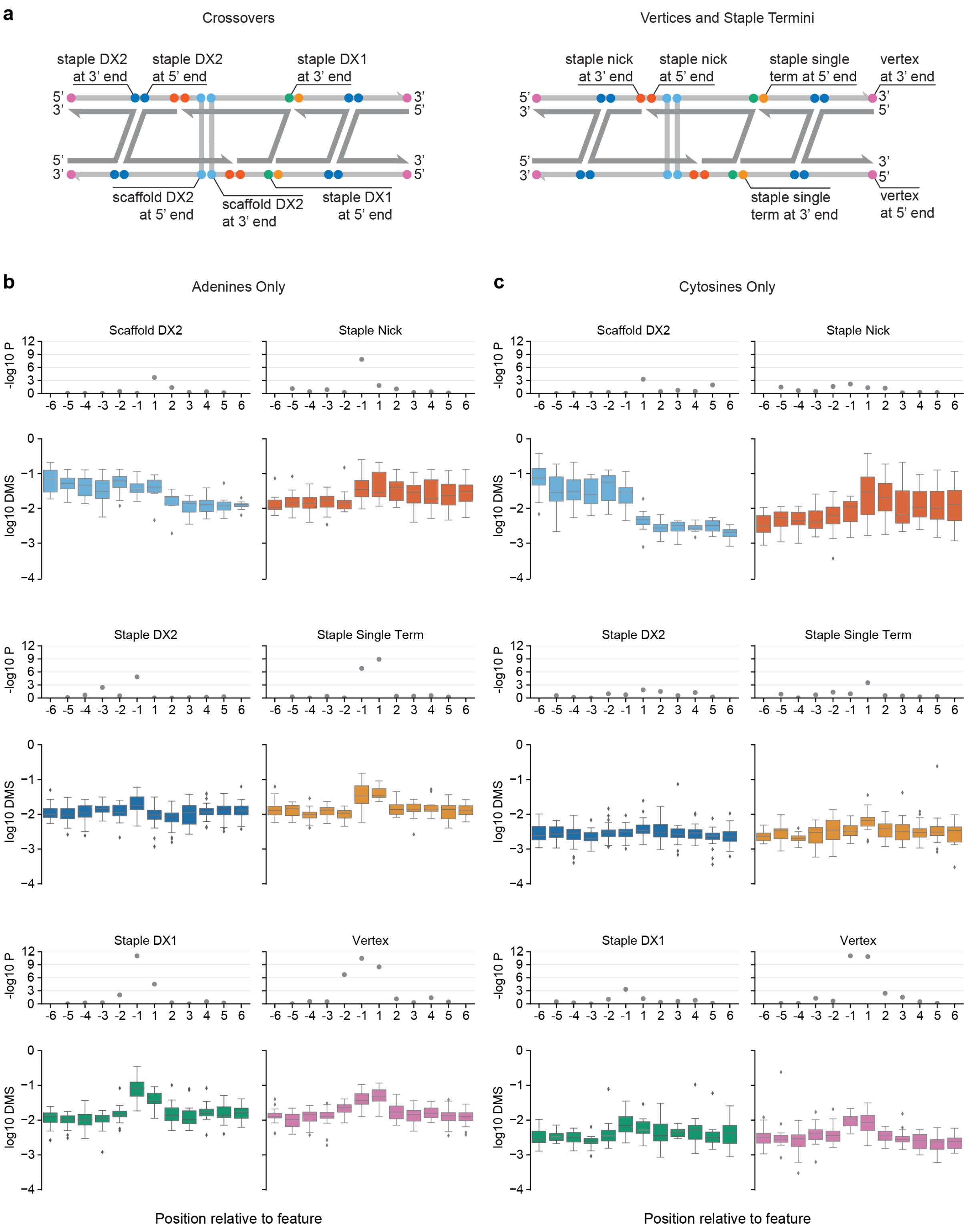

Supplementary Figure 26. **Comparison of per-nucleotide stabilities on adenines and cytosines. a** Schematic of dual-duplex antiparallel crossover (DX) edge comprising RNA scaffold (light grey) and DNA staple (dark grey) strands. Structural features (including those of staples) are labeled on the scaffold (as all DMS-MaPseq data come from the RNA scaffold). DX1: single

crossover, DX2: double crossover, term: terminus, 5' and 3': ends of unbroken segments of RNA:DNA duplex (5'/3' indicates the direction with respect to the scaffold). **b** and **c** Distributions of DMS reactivities among adenines (b) and cytosines (c) within 6 bp of each type of feature in *n* = 5 independent A-form origami (rT55, rT66, rT77, rO66, rPB66; *n* = 1 replicate each). Negative and positive positions lie 5' and 3' of the feature, respectively. Positions -1 and 1, respectively, lie immediately 5' and 3' of the feature and correspond to the 3' and 5' ends of unbroken segments of RNA:DNA duplex, a subset of which are labeled in (a). Each box plot depicts the median (middle line), 1st and 3rd quartiles (box), minimum/maximum up to 1.5 interquartile ranges from box (whiskers), and outliers (grey diamonds). The *P*-value at each position indicates the significance of the median reactivity at that position exceeding the median reactivity among all other positions on the same side of and further from the feature – that is, if the current position is negative, among all of the more negative positions, and if positive, among all of the more positive positions; hence, the *P*-value is undefined for positions ±6 (one-sided Mann-Whitney *U* test). Source data are provided as a Source Data file.

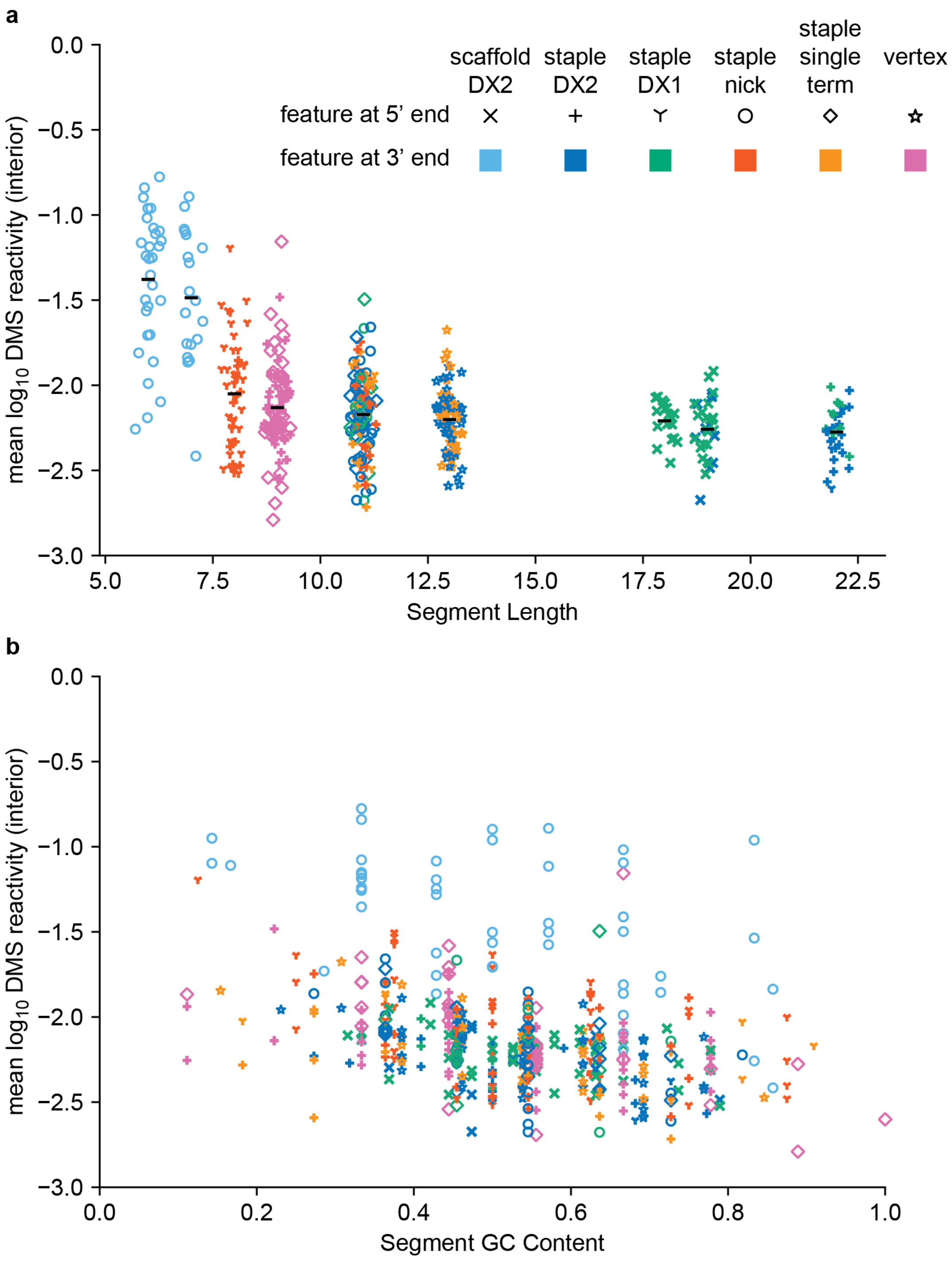

Supplementary Figure 27. **Comparison of mean DMS reactivities among interior (excluding 5’ and 3’ ends) adenines and cytosines with additional segment attributes.** Comparison to **a** lengths of the segments or **b** GC contents of the segments. Segments (n = 490) are pooled from n = 5 independent A-form origami (rT55, rT66, rT77, rO66, rPB66), each with n = 1 replicate. Each point depicts one segment whose 5’ and 3’ end features determine the point’s shape and color, respectively. In (a), jitter has been applied to better visualize points with the same segment lengths, and horizontal black bars indicate the mean log DMS reactivity for each segment length. Source data are provided as a Source Data file.

Supplementary Table 1. **Parameters used in cryo-EM reconstruction for each hybrid nucleic acid origami object.**

Object	Helical geometry design	# Images collected	# Particles used in reconstruction	Symmetry used
rT66	A-form	1070	42216	Tetrahedral
rPB66	A-form	2910	14402	D5
rO44	A-form	828	22340	Octahedral
rO66	A-form	1459	38580	Octahedral
rT66	Alt A-form	314	819	Tetrahedral
rPB66	Alt A-form	1166	7073	D5
rT66	Sym A-form	502	2171	Tetrahedral

Supplementary References

1. Veneziano, R. *et al.* Designer nanoscale DNA assemblies programmed from the top down. *Science* **352**, 1534 (2016).
2. Wang, P., Ko, S. H., Tian, C., Hao, C. & Mao, C. RNA-DNA hybrid origami: folding of a long RNA single strand into complex nanostructures using short DNA helper strands. *Chemical Communications* **49**, 5462–5464 (2013).
3. Zhou, L. *et al.* A mini DNA–RNA hybrid origami nanobrick. *Nanoscale Advances* **3**, 4048–4051 (2021).
