# Supplementary material for: 3D RNA-scaffolded wireframe origami
Source: Nat Commun. 2023 Jan 24;14:382. doi: 10.1038/s41467-023-36156-1 (PMC9872083; doi:10.1038/s41467-023-36156-1)
Supplement: Supplementary file 3 — Description of Additional Supplementary Files [file 41467_2023_36156_MOESM3_ESM.pdf]

Description of Additional Supplementary Files

Supplementary Data 1 – The RNA scaffold sequences used to design each origami, the full RNA sequences used in folding, and their corresponding DNA template sequences.

Supplementary Data 2 – The DNA primer sequences used to amplify the DNA templates in Supplementary Data 1.

Supplementary Data 3 – The DNA staple sequences used to fold each origami object.

Supplementary Data 4 – For each A-form origami probed with DMS-MaPseq, the resultant DMS reactivity of each nucleotide in the scaffold sequence and the feature assignments.
