# Supplementary material for: 3D RNA-scaffolded wireframe origami
Source: Nat Commun. 2023 Jan 24;14:382. doi: 10.1038/s41467-023-36156-1 (PMC9872083; doi:10.1038/s41467-023-36156-1)
Supplement: Supplementary file 8 — Reporting Summary [file 41467_2023_36156_MOESM8_ESM.pdf]

nature portfolio

Corresponding author(s): Mark Bathe

Last updated by author(s): 1/13/2023

Reporting Summary

Nature Portfolio wishes to improve the reproducibility of the work that we publish. This form provides structure for consistency and transparency in reporting. For further information on Nature Portfolio policies, see our Editorial Policies and the Editorial Policy Checklist.

Statistics

For all statistical analyses, confirm that the following items are present in the figure legend, table legend, main text, or Methods section.

n/a	Confirmed	
☐	☒	The exact sample size (*n*) for each experimental group/condition, given as a discrete number and unit of measurement
☐	☒	A statement on whether measurements were taken from distinct samples or whether the same sample was measured repeatedly
☐	☒	The statistical test(s) used AND whether they are one- or two-sided<br>*Only common tests should be described solely by name; describe more complex techniques in the Methods section.*
☐	☒	A description of all covariates tested
☐	☒	A description of any assumptions or corrections, such as tests of normality and adjustment for multiple comparisons
☐	☒	A full description of the statistical parameters including central tendency (e.g. means) or other basic estimates (e.g. regression coefficient) AND variation (e.g. standard deviation) or associated estimates of uncertainty (e.g. confidence intervals)
☐	☒	For null hypothesis testing, the test statistic (e.g. *F*, *t*, *r*) with confidence intervals, effect sizes, degrees of freedom and *P* value noted<br>*Give P values as exact values whenever suitable.*
☒	☐	For Bayesian analysis, information on the choice of priors and Markov chain Monte Carlo settings
☒	☐	For hierarchical and complex designs, identification of the appropriate level for tests and full reporting of outcomes
☐	☒	Estimates of effect sizes (e.g. Cohen's *d*, Pearson's *r*), indicating how they were calculated

Our web collection on statistics for biologists contains articles on many of the points above.

Software and code

Policy information about availability of computer code

Data collection	For cryo-electron microscopy, data were collected using EPU 2.7 software (Thermo Fisher Scientific).
Data analysis	Source code for pyDAEDALUS is available for download at https://github.com/lcbb/pyDAEDALUS.<br>Cryo-electron microscopy data were analyzed using the open-source softwares EMAN v2.3, MotionCor2 v1.3.0, and UCSF Chimera v1.14.<br>DMS-MaPseq sequencing data were analyzed using the open-source software DREEM v1.0 and with custom codes available at https://github.com/lcbb/3dRnaScaffoldedWireframeOrigami, https://github.com/lcbb/ariadne, and https://github.com/lcbb/rouls. The custom codes have the following software dependencies: Python 3.8, NumPy 1.20, Pandas 1.2, Matplotlib 3.3, Seaborn 0.10, BioPython 1.7, and SciPy 1.6.

For manuscripts utilizing custom algorithms or software that are central to the research but not yet described in published literature, software must be made available to editors and reviewers. We strongly encourage code deposition in a community repository (e.g. GitHub). See the Nature Portfolio guidelines for submitting code & software for further information.

Data

Policy information about availability of data

All manuscripts must include a data availability statement. This statement should provide the following information, where applicable:

- Accession codes, unique identifiers, or web links for publicly available datasets
- A description of any restrictions on data availability
- For clinical datasets or third party data, please ensure that the statement adheres to our policy

The electron density maps from cryo-EM generated in this study have been deposited in the EMDB repository under accession codes EMD-34058, EMD-34059, EMD-34060, and EMD-34061. The sequence alignment maps from DMS-MaPseq generated in this study have been deposited in the NCBI Sequence Read Archive under accession code PRJNA868816. A table of all DMS reactivities and structural features of every origami is provided in Supplementary Data 4. All other data generated or analyzed during this study are included in this article, its Supplementary Information and Supplementary Data files. Source data are provided with this paper.

Human research participants

Policy information about studies involving human research participants and Sex and Gender in Research.

Reporting on sex and gender	This information has not been collected--no human research participants were involved int his study.
Population characteristics	This information has not been collected--no human research participants were involved int his study.
Recruitment	No recruitment was done--no human research participants were involved in this study.
Ethics oversight	No approval was required--no human research participants were involved in this study.

Note that full information on the approval of the study protocol must also be provided in the manuscript.

Field-specific reporting

Please select the one below that is the best fit for your research. If you are not sure, read the appropriate sections before making your selection.

☒ Life sciences ☐ Behavioural & social sciences ☐ Ecological, evolutionary & environmental sciences

For a reference copy of the document with all sections, see nature.com/documents/nr-reporting-summary-flat.pdf

Life sciences study design

All studies must disclose on these points even when the disclosure is negative.

Sample size	Statistical tests were applied only to DMS-MaPseq data. The number of origami per replicate was determined by the number of A-form origami we tested (n = 5). The numbers of double helical segments and of A/C nucleotides per origami were determined by pyDAEDALUS using the sequence and target geometry of each origami. We first performed and analyzed one replicate of every origami and found that sets of DMS reactivities expected to be similar (see "Replication" below) were highly correlated, and that sets expected to differ did so at a significance level of 0.01. Thus we concluded that we did not need to increase the sample size by performing additional replicates.
Data exclusions	None of the DMS-MaPseq data from the n = 5 A-form origami were excluded. DMS-MaPseq was also performed on n = 4 Alt A-form versions of rT66, rO44, rO66, and rPB66, originally with the intention of comparing them to their A-form counterparts. Later on, inspection of the predicted atomic structures indicated that the A-form design yielded less steric strain than the Alt A-form design, so we refocused the manuscript on the A-form origami. Because we discuss the Alt A-form origami only briefly in the main text and in the Supplementary Information, we decided that the DMS-MaPseq data for the n = 4 Alt A-form designs were extraneous and thus excluded them from analysis.
Replication	Although we analyzed only n = 1 replicate of each origami with cryo-EM and DMS-MaPseq, we were able to compare regions of different origami objects that we expected to be similar. Namely, the DMS reactivities of rT66 folded with versus without staple 10 were highly correlated (r = 0.91) outside of the region targeted by staple 10, as were the DMS reactivities over the last ~400 nt of rO66 (which were not targeted by staples) and the 23S scaffold folded without staples (r = 0.89). In the aforementioned experiments, each origami was folded and probed with DMS-MaPseq independently. From these findings of n = 1 independent experiment for each origami, we concluded that the DMS-MaPseq data were reproducible.
Randomization	Randomization was not relevant to this study because different origamis were not assigned to different experimental or treatment groups; rather, the differences originated in the design of each origami.
Blinding	Blinding was not performed in this study because there were no samples assigned to random groups.

Reporting for specific materials, systems and methods

We require information from authors about some types of materials, experimental systems and methods used in many studies. Here, indicate whether each material, system or method listed is relevant to your study. If you are not sure if a list item applies to your research, read the appropriate section before selecting a response.

Materials & experimental systems

n/a	Involved in the study
☒	☐ Antibodies
☒	☐ Eukaryotic cell lines
☒	☐ Palaeontology and archaeology
☒	☐ Animals and other organisms
☒	☐ Clinical data
☒	☐ Dual use research of concern

Methods

n/a	Involved in the study
☒	☐ ChIP-seq
☒	☐ Flow cytometry
☒	☐ MRI-based neuroimaging
